# Microstructural Abnormalities in Subcortical Reward Circuitry of Subjects with Major Depressive Disorder

**DOI:** 10.1371/journal.pone.0013945

**Published:** 2010-11-29

**Authors:** Anne J. Blood, Dan V. Iosifescu, Nikos Makris, Roy H. Perlis, David N. Kennedy, Darin D. Dougherty, Byoung Woo Kim, Myung Joo Lee, Shirley Wu, Sang Lee, Jesse Calhoun, Steven M. Hodge, Maurizio Fava, Bruce R. Rosen, Jordan W. Smoller, Gregory P. Gasic, Hans C. Breiter, for the Phenotype Genotype Project on Addiction and Mood Disorders

**Affiliations:** 1 Depression Clinic and Research Program, Mood and Motor Control Laboratory, Addiction Research Program, Laboratory of Neuroimaging and Genetics, Department of Psychiatry, Massachusetts General Hospital and Harvard Medical School, Boston, Massachusetts, United States of America; 2 Psychiatric and Neurodevelopmental Genetics Unit and Center for Human Genetic Research, Massachusetts General Hospital and Harvard Medical School, Boston, Massachusetts, United States of America; 3 Motivation and Emotion Neuroscience Collaboration (MENC) and Athinoula A. Martinos Center for Biomedical Imaging, Department of Radiology, Massachusetts General Hospital and Harvard Medical School, Boston, Massachusetts, United States of America; 4 Center for Morphometric Analysis and Center for Integrative Informatics, Massachusetts General Hospital and Harvard Medical School, Boston, Massachusetts, United States of America; 5 Department of Neurology, Massachusetts General Hospital and Harvard Medical School, Boston, Massachusetts, United States of America; 6 Mount Sinai School of Medicine, New York, New York, United States of America; University of Minnesota, United States of America

## Abstract

**Background:**

Previous studies of major depressive disorder (MDD) have focused on abnormalities in the prefrontal cortex and medial temporal regions. There has been little investigation in MDD of midbrain and subcortical regions central to reward/aversion function, such as the ventral tegmental area/substantia nigra (VTA/SN), and medial forebrain bundle (MFB).

**Methodology/Principal Findings:**

We investigated the microstructural integrity of this circuitry using diffusion tensor imaging (DTI) in 22 MDD subjects and compared them with 22 matched healthy control subjects. Fractional anisotropy (FA) values were increased in the right VT and reduced in dorsolateral prefrontal white matter in MDD subjects. Follow-up analysis suggested two distinct subgroups of MDD patients, which exhibited non-overlapping abnormalities in reward/aversion circuitry. The MDD subgroup with abnormal FA values in VT exhibited significantly greater trait anxiety than the subgroup with normal FA values in VT, but the subgroups did not differ in levels of anhedonia, sadness, or overall depression severity.

**Conclusions/Significance:**

These findings suggest that MDD may be associated with abnormal microstructure in brain reward/aversion regions, and that there may be at least two subtypes of microstructural abnormalities which each impact core symptoms of depression.

## Introduction

It has been proposed that major depressive disorder (MDD) may result from dysfunction of brain reward/aversion circuitry[1], [2], [3], [4], [5], [6], [7]. Hypothesized in 1999 as a general schema for processing both positive and negative features of potential “goal-objects” or states [8], a generalized reward/aversion system that processes the salience of stimuli across a continuum of aversion and reward was described in multiple publications between 1996 and 2001 [9], [10], [11], [12], [13]. This work identified an extended set of brain regions as variably processing a continuum between positive and negative valence and intensity information, along with category and incidence information from goal-objects. These observations have been extensively replicated [14], [15], [16], [17], [18] and synopsized [19], [20], [21]. The recent report of a law-like relationship between patterns of approach and avoidance behavior to rewarding and aversive stimuli further argues that the systems processing this information do not function independently of one another [22].

The possibility that abnormalities in this reward/aversion circuitry underlie many psychiatric conditions, including MDD, was further schematized by multiple investigators [1], [6], [23], [24]. Animal models of MDD strongly support this thesis and have hypothesized dopaminergic midbrain nuclei and the medial forebrain bundle (MFB) to be involved with the illness [7], [25]; these subcortical regions are at the core of the animal literature that first identified reward circuitry [26], [27], [28], [29], [30]. In humans with MDD, there has recently been detection of functional abnormalities in these subcortical regions [2], [31], [32], in addition to well-established abnormalities in target regions receiving subcortical projections [33], [34], [35], [36], [37], [38], [39], [40], [41], [42], [43], [44], [45], [46], [47]. However, there has not yet been an evaluation of microstructural integrity in the midbrain nuclei or MFB.

The current study aimed to evaluate microstructural integrity of subcortical brain reward/aversion circuitry in cohorts of subjects with and without MDD, using approaches that maximized detection sensitivity in subcortical regions. Our primary hypotheses focused on evaluating brain microstructure in the midbrain ventral tegmental area/substantia nigra (VTA/SN), the medial forebrain bundle (MFB), and the amygdalofugal pathway (AFP). Our secondary *a priori* hypotheses were based on a general evaluation of the broad set of reward/aversion circuitry implicated in MDD [8], [48].

We designed the study to take into consideration potential heterogeneity within the MDD cohort [33], [37], [49], [50], [51], [52], [53], [54], [55], [56], [57], given that this is a potential confound in group designs [58]. Such an approach may not only improve methods, but may also lead to identification of subgroups of MDD. We hypothesized there would be microstructural heterogeneity in our cohort that could not be predicted *a priori* by functional imaging abnormalities or symptom profiles since MDD has been hypothesized to be a systems-level disorder, and it is possible that microstructural abnormalities at different points in a distributed circuit could all lead to similar abnormalities in behavior/mood. To address this, we allowed the DTI data itself to drive potential segregation across patients.

## Methods

### Subjects

All subjects signed written informed consent prior to participation in this study, and the study was approved by the Institutional Review Board of Massachusetts General Hospital (Partners Human Research Committee). All experiments were conducted in accordance with the principles of the Declaration of Helsinki.

22 of 44 subjects met DSM-IV criteria for Major Depressive Disorder (MDD) diagnosed by physician-administered Structured Clinical Interview for DSM-IV Axis I Disorders - Patient Edition [SCID-P [59]] and were between the ages of 18 and 65 (mean age = 36.3±12.1 years; 12/22 females, mean educational history of 15.6±2.6 years, 19 Caucasian and 3 African American, 20/22 right-handed). These patients were matched one-to-one with 22 healthy volunteers on the following criteria: age (within 5 years), years of education (within 5 years), gender, self-reported race, and handedness (control mean age = 35.3±11.6 years; mean educational history of 15.7±2.1 years; 12/22 females, 19 Caucasian and 3 African American, 20/22 right-handed. Age and years of education did not differ significantly across groups (age: F = 0.001, p = 0.979;.education: F = 0.085, p = 0.772). This tight matching was done because each of these factors may potentially influence neural structure and function. All subjects were drawn from a larger study evaluating cocaine addicted, depressed and control subjects [The Massachusetts General Hospital (MGH) Phenotype Genotype Project in Addiction and Depression (PGP; http://pgp.mgh.harvard.edu)]. The 22 MDD subjects were drawn from a larger cohort of individuals with MDD (n = 47) because they met quality assurance criteria, including (a) minimal residual motion artifacts after motion correction of DTI images, (b) absence of MR susceptibility artifacts, and (c) availability of control subjects which met the strict matching criteria described above, and who also had DTI data without motion or susceptibility artifacts.

MDD subjects were excluded if they met DSM-IV criteria for primary psychotic disorders, bipolar disorder, eating disorders, substance abuse disorders, generalized anxiety disorder, panic disorder, PTSD or OCD by SCID interview (current or lifetime); healthy volunteers were excluded if they met DSM-IV criteria for any Axis I psychiatric disorder by SCID interview.

Additional exclusion criteria for both MDD subjects and healthy volunteers were: 1) currently suicidal or at risk for suicide in the judgment of the investigator; 2) pregnant women; 3) carrying a medical device incompatible with MRI (e.g., metal implants such as surgical clips or pacemakers) or significant claustrophobia or weight that would make MRI unfeasible; 4) serious medical illness including a known history of HIV-1+ status; 5) Subjects with insulin dependent diabetes mellitus (IDDM) or subjects with noninsulin dependent diabetes mellitus (NIDDM) and abnormal Hemoglobin A1C; 6) severe respiratory compromise; 7) history of head trauma with neurological sequelae, including multiple concussions and/or history of stroke; 8) history of seizure disorder, delirium, dementia, or mental disorders due to general medical conditions; 9) clinical or laboratory evidence of uncontrolled hypothyroidism or hyperthyroidism; and 10) subjects which, in the opinion of the Management Group running the PGP, were not able to participate safely in this study. In addition, subjects in the larger PGP study were screened for Hepatitis C (by Hepatitis C+ titer); no subjects included in this study tested positive for Hepatitis C. Three MDD subjects and one control subject had a history of tobacco use (one current smoker in MDD cohort, one current smoker in the control cohort, and two previous smokers in the MDD cohort).

In addition to screening for exclusion criteria, we also performed for all subjects (MDD and healthy controls): (1) the Structured Clinical Interview for DSM-IV for Axis I disorders (SCID-I); (2) a medical history and concurrent medication status (see Table 1 for medication status in relation to MDD subgrouping and VTA/SN FA values); (3) sociodemographic information; (4) Edinburgh Handedness assay; (5) blood and urinary analysis; (6) Inventory of Depressive Symptomatology – Self Report (IDS-SR); (7) 31-item Hamilton Rating Scale-Depression (HAM-D, [60]); and (8) the STAI (Spielberger State Trait Anxiety Inventory).

**Table 1:** Antidepressant History and Status for MDD Cohort.

FA value in VTA/SN cluster	History of treatment with antidepressants?	On antidepressant(s) at time of scanning?
**Normal VTA/SN subgroup**		
0.263588	No	No
0.332777	Yes	Yes
0.353066	Yes	Yes
0.362028	Yes	No
0.390219	No	No
0.39181	Yes	No
0.410209	Yes	Yes
0.417723	No	No
0.434277	No	No
0.43575	No	No
0.448794	Yes	Yes
0.455736	Yes	No
**Abnormal VTA/SN subgroup**		
0.544226	Yes	No
0.546144	No	No
0.54869	Yes	No
0.602679	Yes	Yes
0.603839	No	No
0.612006	Yes	No
0.646446	Yes	Yes
0.658727	Yes	Yes
0.67705	No	No
0.745329	Yes	No

Antidepressant history and current status were determined during study screening based on the SCID evaluation (history) and a questionnaire reporting current medication status. A yes response indicates a positive history and/or current usage with an identified antidepressant medication and dosage.

### DTI Scanning Protocol

During each scanning session, a high-resolution (2 mm isotropic) whole head DTI scan was acquired on a Siemens 3.0 Tesla Sonata Magnet System (Siemens AG, Medical Solutions, Erlangen, Germany); TR = 24 s; TE = 81 ms; slice thickness = 2 mm, 60 slices total, 128×128 matrix, 256×256 mm FOV, 6 averages, 6 noncolinear directions with b value = 700 s/mm^2^, and 1 image with b-value = 0 s/mm^2^. DTI scans were acquired using auto-align software [61] to normalize brain image slice orientation across subjects. Slices were situated parallel to the AC-PC line, and parallel to the inside curve of the FOC to minimize signal distortion in this region [62].

### DTI Image Preprocessing and Registration

#### 1. DTI preprocessing

All data processing was performed using Freesurfer software (http://surfer.nmr.mgh.harvard.edu) and FSL (http://www.fmrib.ox.ac.uk/fsl) processing streams. Detailed methods for preprocessing DTI data are described in Salat et al. [63]. Briefly, each tensor volume from the DTI dataset was resampled to the T2 image to correct for remaining eddy current distortion, and to correct for head motion. The fractional anisotropy (FA) metric was derived from the diffusion tensor as previously described [64]. All resulting maps were resampled to 1 mm^3^ resolution. Images were inspected for residual motion and susceptibility artifacts; subjects with significant artifacts were excluded.

#### 2. Image registration

FA maps were registered to a Montreal Neurological Institute (MNI) ICBM152 T2 template for the voxel-based contrast and subsequent analyses; all contrast analysis results are reported in MNI coordinates. Registration procedures (both automated and manual) were done blinded to subject and group identification. Given the small size of our areas of interest, our methods included registration techniques designed to maximize intersubject alignment in our areas of interest. Registration and analysis of the VTA/SN, in particular, has been previously shown to be valid and informative, given abnormalities have been detected in the SN in patients with Parkinson's Disease, a population known to exhibit structural pathology in this region [65], [66]. In PD patients, FA was decreased in the SN, which is consistent with neuronal loss in this region [65], [66]. While the Parkinson's studies did not use our directed registration methods, the abnormalities in these patients (i.e. the “signal”) was presumed to be much greater in Parkinson's patients, making it less susceptible to being masked by imprecision in registration of small nuclei by automated methods (i.e. “noise”). Thus, in a disorder where we expect more subtle abnormalities in this region (e.g., MDD), we believe the directed registration methods were required to resolve the signal above the noise.

*a. Automated registration*. The initial image registration was done using FSL software to perform an automated, 12-degrees of freedom, global affine transformation[67]. The linear affine transformation (in combination with a subsequent landmark-guided manual registration) was selected over non-linear transform methods to minimize loss of signal due to warping, which is relatively greater in the small subcortical regions [68] relevant to our *a priori* hypotheses.

*b. Manual registration*. After the automated registration, all registered images were put through an additional manual registration step using Martinos Center Freesurfer software (http://surfer.nmr.mgh.harvard.edu). This step maximized the accuracy of registration of each subject's FA map to ICBM152 T2 space, based on three registration landmarks selected to maximize registration in our *a priori areas of evaluation* (AOEs) (see Text S1). Figures S1, S2, S3, S4 illustrate anatomical co-localization across MDD and control cohorts overall, and within our primary AOEs.

### Data Analysis

#### 1. A priori hypotheses and segmentation of a priori areas of evaluation (AOEs)

Primary and secondary *a priori* hypotheses corresponded to *a priori* AOEs for constraining our voxel-based search of patient/control differences. These AOEs constrained which voxels were evaluated in FA map group contrasts, to identify clusters meeting volume and significance thresholds. AOEs were segmented by an anatomist (N.M.) using landmark-based, atlas-guided definitions of the regions (see Text S1 and Figures S1, S2, S3, S4, S5, S6, S7).

*a. Primary a priori hypothesis*. We predicted MDD subjects would exhibit abnormal brain microstructure in (1) the medial forebrain bundle (MFB) and contiguous lateral nucleus of the hypothalamus (LNH), along with (2) regions feeding the MFB/LNH, including the ventral tegmental area/substantia nigra (VTA/SN), and (3) the amygdalofugal pathway. Follow-up analyses (described below), considered clusters falling within these regions.

*b. Secondary a priori hypotheses*. Based on imaging findings in the MDD literature, we further hypothesized that MDD subjects would exhibit abnormal brain microstructure in white matter adjacent to orbitofrontal cortex (FOC), anterior cingulate cortex (ACC), paracingulate cortex (PAC), subgenual prefrontal cortex (SGC), and dorsolateral prefrontal cortex (DLPFC).

*c. Brain regions outside a priori hypothesized AOEs*. We evaluated all other brain regions outside primary and secondary *a priori* AOEs using a whole-brain Bonferroni correction.

#### 2. Voxel-based contrast of FA maps for MDD subjects versus control subjects

*a. Contrast analysis*. A voxel-based contrast analysis was performed between MDD and control subjects, using a two-tailed t-test with Freediffusion software [63], [69]. To minimize the chance of false positives we required (1) that group differences meet a cluster threshold and (2) that the p value of the peak voxel within each cluster meet a correction for the number of voxels in a search volume.

*b. Cluster thresholds for group contrast of FA maps*. For primary *a priori* AOEs, the cluster threshold was at least 9 contiguous voxels, with p<0.05 for each voxel. This was increased to 27 contiguous voxels for secondary *a priori* AOEs, and 81 contiguous voxels for regions outside *a priori* AOEs. The least stringent threshold exceeded cluster thresholds used with fMRI [30], [70] and cortical thickness analysis[71]. We defined contiguous voxels as voxels sharing an edge (i.e., not just a corner). To be considered within that AOE, greater than 50% of voxels in a cluster had to fall within a segmented *a priori* AOE.

*c. Multiple comparisons correction for group contrast of FA maps*. To be considered statistically significant, clusters were required to have a peak voxel meeting a Bonferroni correction for multiple comparisons. Corrections were based on the total number of voxels in the area being considered (418 for primary *a priori* AOEs; 9,308 for secondary *a priori* AOEs; 200,000 for the whole brain [i.e., not *a priori*]), divided by the required cluster size. Thus, the uncorrected p value (reported in Tables 2, 3, 4) was required to be p<0.05/(418/9), or p<0.00108, for primary *a priori* AOEs, p<0.05/(9,308/27), or p<1.45×10^−4^, for secondary *a priori* AOEs, and p<0.05/(200,000/81), or 2.03×10^−5^, for the rest of the brain. Peak voxels within an order of magnitude of the corrected p value were considered trends toward significance. All regions that met cluster threshold criteria were tabulated; however, the results and discussion sections focus primarily on regions which met full significance criteria.

**Table 2:** Voxel-Based Contrast for the Whole Cohort (22 MDD Versus 22 Matched Controls).

Region	MNI coordinates at peak difference	t (p) values at peak difference	Cluster size(# voxels)
**FA group differences which met the 9-voxel cluster requirement in the primary ** ***a priori*** ** areas of evaluation (AOEs)**			
R. VTA/SN	11.5 −19.2 −13.6	3.56 (0.000929)*	17
L. MFB/LNH	−9.5 −3.9 −11.8	−3.20 (0.00264)†	9
**FA group differences which met the 27-voxel cluster requirement in the secondary ** ***a priori*** ** AOEs**			
R. ACC wm	19.2 32.7 −4.7	−3.79 (0.000472)†	35
R. DLPFC wm (superior fr. gyrus)	18.3 26.5 49.5	−4.00 (0.000252)†	27
L. DLPFC wm (superior fr. gyrus)	−13.3 23.1 44.1	−4.01 (0.000245)†	55
L. DLPFC wm (middle fr. gyrus)	−38.6 26.0 31.5	−3.20 (0.00262)	40
L ACC/PAC wm	−9.5 38.5 17.0	−3.49 (0.00113)	33
**FA group differences which met the 81-voxel cluster requirement in other regions**			
L. PMC wm (SLF3)	−43.9 0.0 18.9	−4.50 (0.000052)†	81
R. PMC wm (SLF3)	45.5 0.0 17.0	−4.15 (0.000160)	134
L./midline CC	−4.7 5.3 22.5	−3.56 (0.000937)	98
R. CCtx wm	30.6 −57.5 2.6	5.54(0.0000018)*	83
L. CCtx wm	−21.0 −60.1 2.7	4.29 (0.000102)	99

Positive t values indicate FA values were elevated in MDD subjects relative to control subjects; negative t values indicate FA values were reduced in MDD subjects relative to control subjects. p values are reported uncorrected; symbols indicate significance and trends at the corrected threshold.

*p value met the corrected threshold.

†p value was within an order of magnitude of the corrected threshold (a trend).

Abbreviations: R: right hemisphere; L: left hemisphere; wm: white matter; gm: gray matter; VTA/SN: ventral tegmental area/substantia nigra; MFB: medial forebrain bundle; LNH: lateral nucleus of the hypothalamus; ACC anterior cingulate cortex; DLPFC: dorsolateral prefrontal cortex; PAC: paracingulate cortex; PMC: premotor cortex; CC: corpus callosum; CCtx: calcarine cortex.

**Table 3:** Post-Hoc Contrast for “Abnormal VTA/SN” MDD Subgroup (10 MDD Versus 10 Matched Controls).

Region	MNI coordinates at peak difference	t (p) values at peak difference	Cluster size (# voxels)
**FA group differences which met the 9-voxel cluster requirement in the primary ** ***a priori*** ** areas of evaluation (AOEs)**			
R. VTA/SN	11.4 −21.1 −11.9	6.62 (0.0000032)*	49
L. VTA/SN	−5.7 −19.2 −17.2	3.90 (0.00106)*	13
**FA group differences which met the 27-voxel cluster requirement in the secondary ** ***a priori*** ** AOEs**			
L. DLPFC wm (superior fr. gyrus)	−15 26.9 32.8	−3.31 (0.00394)	50
L. DLPFC wm (middle fr. gyrus)	−37.7 27.5 27.7	−3.82 (0.00127)	33
**FA group differences which met the 81-voxel cluster requirement in other regions**			
R. FOC (olf sulcus) gm	9.5 17.9 −15.4	4.96 (0.000102)	95
R. SGC gm	7.6 10.7 −5.8	4.63 (0.000210)	118
R. PMC wm (SLF3)	53 9.2 15.2	−5.95 (0.000012)*	106
MDM/PAG region	0 −24.6 −7.2	4.26 (0.000471)	78 (just below significance)

Positive t values indicate FA values were elevated in MDD subjects relative to control subjects; negative t values indicate FA values were reduced in MDD subjects relative to control subjects. p values are reported uncorrected; symbols indicate significance and trends at the corrected threshold.

*p value met the corrected threshold.

†p value was within an order of magnitude of the corrected threshold (a trend).

Abbreviations: R: right hemisphere; L: left hemisphere; wm: white matter; gm: gray matter; VTA/SN: ventral tegmental area/substantia nigra; DLPFC: dorsolateral prefrontal cortex; FOC: orbitofrontal cortex; SGC: subgenual cortex; PMC: premotor cortex; CCtx: calcarine cortex; MDM/PAG: medial dorsal midbrain/periaqueductal gray.

**Table 4:** Post-Hoc Contrast for “Normal VTA/SN” MDD Subgroup (12 MDD Versus 12 Matched Controls).

Region	MNI coordinates at peak difference	t (p) values at peak difference	Cluster size (# voxels)
**FA group differences which met the 9-voxel cluster requirement in the primary ** ***a priori*** ** areas of evaluation (AOEs)**			
L. MFB/LNH	−11.3 −3.3 −9.8	−3.19 (0.00425)†	20
**FA group differences which met the 27-voxel cluster requirement in the secondary ** ***a priori*** ** AOEs**			
R. ACC wm	15.2 31.6 −3.2	−3.83 (0.000913)†	31
L ACC/PAC wm	−9.4 38 13.8	−3.56 (0.00175)	46

Positive t values indicate FA values were elevated in MDD subjects relative to control subjects; negative t values indicate FA values were reduced in MDD subjects relative to control subjects. p values are reported uncorrected; symbols indicate significance and trends at the corrected threshold.

*p value met the corrected threshold.

†p value was within an order of magnitude of the corrected threshold (a trend).

Abbreviations: R: right hemisphere; L: left hemisphere; wm: white matter; MFB: medial forebrain bundle; LNH: lateral nucleus of the hypothalamus; ACC anterior cingulate cortex; PAC: paracingulate cortex.

*d. Permutation test as validation of our findings*. As a supplemental test to further validate the robustness of findings, we computed a permutation test for regions meeting the cluster threshold. Methods and results for this analysis are reported in Tables S1, S2, S3, S4, S5, S6 in Dataset S1.

*e. Laterality test*. Given initial findings in the VTA/SN region were lateralized to the right hemisphere in MDD subjects, we assessed laterality of FA in this region for both groups to distinguish between potential presence of lateralized effects in patients, versus potential loss of laterality relative to controls (see Dataset S2).

#### 3. Follow-up analyses

Follow-up analyses were conducted to assess MDD group heterogeneity, and the relationship between FA and symptom profiles for MDD subjects.

*a. Individual subject FA values*. Once clusters representing group differences were identified in primary *a priori* AOEs, we measured mean FA across each cluster for each subject (see Figures S5, S6 for illustrations of these clusters). Mean FA values for individuals were then used in follow-up analyses. To evaluate whether MDD cohort heterogeneity led to false negatives, we included findings which met the cluster, but not the Bonferroni threshold in the initial voxel-based contrast.

*b. Gapping analysis for individual FA values in MDD subjects*. We used a gapping analysis [72] to test the likelihood that there were gaps in the distribution of FA values in the MDD cohort, suggesting the presence of two or more population distributions of these values. This analysis involved rank ordering individual VTA/SN FA values and calculating the mean gap distance between each of the middle 50% of values (eliminating the top 25% and bottom 25% of values to exclude potential outliers). The ratio of each individual gap distance between adjacent data points was calculated relative to mean gap distance. Finally, we identified the greatest gap ratio within the middle 50% of values and calculated the likelihood that this gap ratio would be observed by chance if data points reflected the distribution of a single population. This likelihood was calculated both for a Gaussian distribution and for a t distribution with df = 4. Each statistic was calculated using 10,000 simulations.

*c. Voxel-based contrasts of FA maps for the two MDD subgroups*. A voxel-based contrast was calculated for each of the two subgroups of MDD subjects identified in the gapping analysis versus their individually matched controls. Clusters were identified in these voxel-based contrasts and peak voxels corrected for multiple comparisons using the same criteria as in the initial group contrast.

*d. VTA/SN FA and symptom measures*. We evaluated whether symptom profiles differed across the two MDD subgroups. Specifically, we looked at (1) overall depression severity (total score on IDS-SR), and scores on individual IDS-SR items relating to (2) anhedonia (question 21), (3) sadness (question 5), and (4) psychomotor symptoms (question 30). Although MDD subjects in our cohort had not been clinically subtyped, anxious depression is one of the most common clinical subtypes of MDD [57], [73]; therefore we also evaluated measures of (5) trait anxiety (via the STAI-T). We used two-tailed t-tests to evaluate whether these measures differed between MDD subgroups. Significance was determined using a Bonferroni correction of p<0.05/5 = 0.01 for these analyses.

## Results

### 1. Image Contrast Analysis of MDD Versus Control Subjects

#### a. Primary a priori areas of evaluation (AOEs) (Table 2; Figure 1)

**Figure 1:**
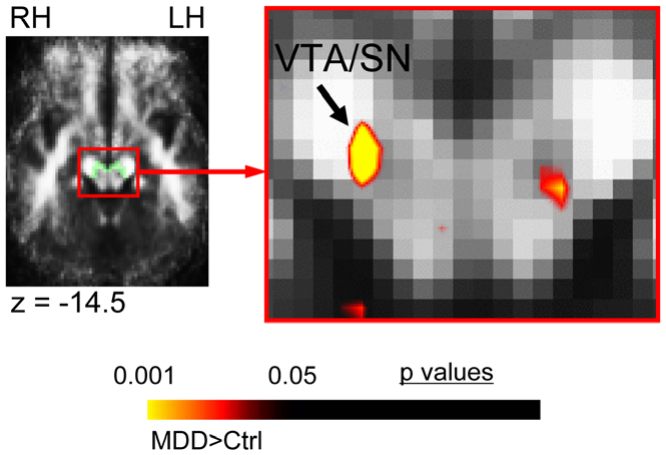
FA difference in MDD subjects in primary *a priori* areas of evaluation. This figure shows the significant FA difference in MDD subjects versus matched healthy controls for the voxel-based contrast (all subjects: 22 MDD, 22 controls) within areas of evaluation (AOEs) included in our primary *a priori* hypotheses. MDD subjects exhibited elevated FA in the ventral tegmental area/substantia nigra (VTA/SN), which localized to the ventral/lateral edge of the substantia nigra (SN) adjacent to the cerebral peduncle, and the nigral fiber system (striatonigral, nigrostriatal, and corticostriatal fibers). Translucent green in image on left indicates the *a priori* AOE for the VTA/SN in the slice shown, which was used to constrain the initial contrast analysis. The color bar indicates the range of p values in this figure, from the threshold (p<0.05) to the order of magnitude of the voxel of peak significance (i.e. smallest p value) in the VTA/SN; color in images is viewed using trilinear interpolation. Warm tones (red, orange, yellow) indicate regions in which MDD subjects exhibited elevated FA relative to control subjects. RH: right hemisphere; LH: left hemisphere.

Significantly elevated FA was detected in MDD subjects within the VTA/SN. Specifically, FA was elevated at the ventral/lateral edge of the right SN adjacent to the cerebral peduncle and overlying the nigral fiber system (striatonigral, nigrostriatal, and corticostriatal fibers; Figure 1). A trend toward reduced FA in MDD subjects was also noted in the left MFB where it passes through the LNH. There were no group differences for the amygdalofugal AOE. Mean FA values for each cluster detected in the voxel-based contrast are reported in Table S2.

#### b. Secondary a priori AOEs (Table 2; Figure 2)

**Figure 2:**
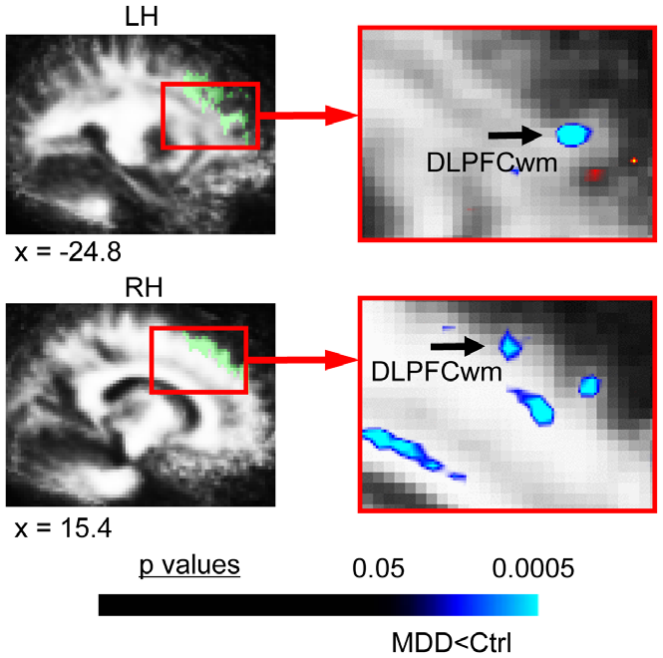
FA differences in MDD subjects in secondary *a priori* areas of evaluation. This figure shows the significant FA differences in MDD subjects versus matched healthy controls for the voxel-based contrast (all subjects: 22 MDD, 22 controls) in *a priori* regions of evaluation (AOEs) included in our secondary *a priori* hypotheses. MDD subjects exhibited reduced FA in white matter regions underlying dorsolateral prefrontal cortex (DLPFCwm), bilaterally. Translucent green in images on left indicates the *a priori* AOE for DLPFCwm in the slices shown, which were used to constrain the initial contrast analysis. The color bar indicates the range of p values in this figure, from the threshold (p<0.05) to the order of magnitude of the voxel of peak significance (i.e. smallest p value) in DLPFCwm; color in images is viewed using trilinear interpolation. Cool tones (blues) indicate regions in which MDD subjects exhibited reduced FA relative to control subjects. RH: right hemisphere; LH: left hemisphere.

There were trends toward significantly reduced FA in two white matter regions underlying DLPFC (Figure 2), and in white matter adjacent to the right ACC.

#### c. Other regions meeting the cluster threshold (Table 2; Figure 3)

**Figure 3:**
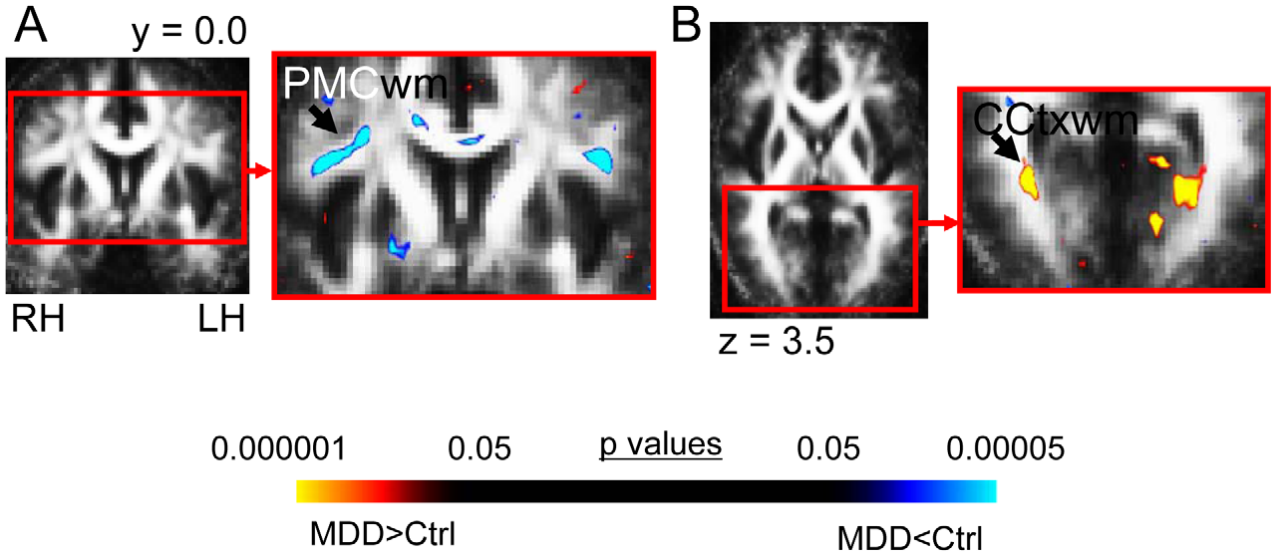
FA differences in MDD subjects in regions not in our a priori areas of evaluation. This figure shows the significant FA differences in MDD subjects versus matched healthy controls for the voxel-based contrast (all subjects: 22 MDD, 22 controls) in regions not included in our *a priori* hypotheses. MDD subjects exhibited (A) reduced FA in white matter adjacent to right premotor cortex (PMCwm), and (B) elevated FA in white matter adjacent to right calcarine cortex (CCtx). The color bar indicates the range of p values in this figure, from the threshold (p<0.05) to the order of magnitude of the voxel of peak significance (i.e. smallest p value) in PMCwm for reduced FA and from the threshold (p<0.05) to the order of magnitude of the voxel of peak significance in CCtx white matter for elevated FA, color in images is viewed using trilinear interpolation. Warm tones (red, orange, yellow) indicate regions in which MDD subjects exhibited elevated FA relative to control subjects; cool tones (blues) indicate regions in which MDD subjects exhibited reduced FA relative to control subjects. RH: right hemisphere; LH: left hemisphere.

Outside *a priori* AOEs, MDD subjects exhibited significantly elevated FA in white matter adjacent to right calcarine cortex (Figure 3B), and a trend toward significantly reduced FA in white matter within the left precentral gyrus, below premotor cortex (BA6) (Figure 3A).

### 2. Follow-up Analyses: Gapping, Subgroup Subtractions, Symptom Correlations

#### a. Gapping analysis

**Figure 4:**
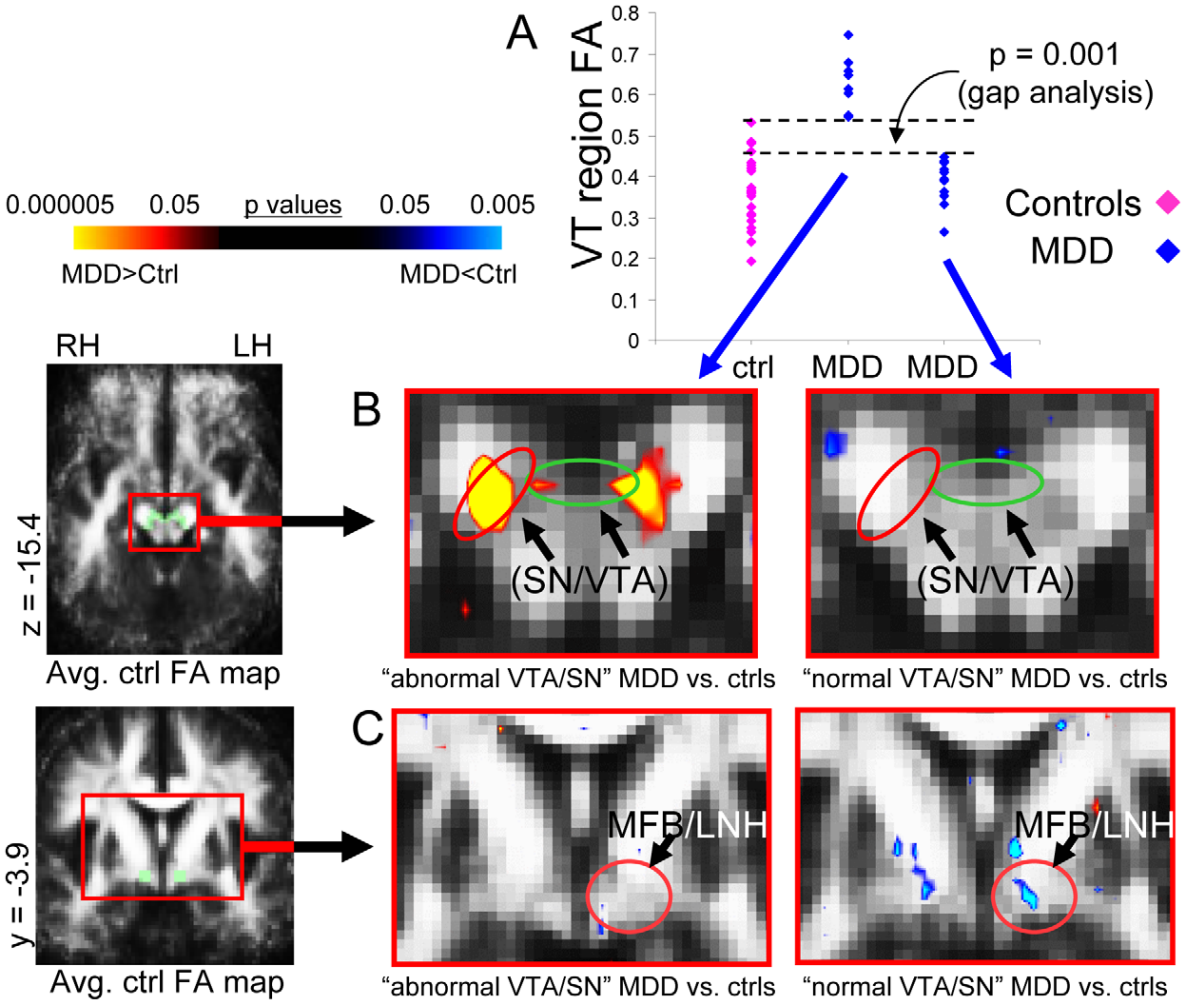
Subgrouping of MDD cohort based on individual FA values. This figure shows the subgrouping of the MDD cohort based on individual FA values, and double dissociation of VTA/SN and MFB abnormalities across these two subgroups. (A) Scatterplots of FA values from the VTA/SN cluster from MDD subjects (blue) and control subjects (pink). There was a statistically significant gap in the middle of the VTA/SN values for MDD subjects; the values above this gap were all outside the range of control values. When contrasts were calculated separately for MDD subjects in the abnormal VTA/SN (10 MDD and 10 controls) versus the normal VTA/SN (12 MDD and 12 controls) MDD subgroups, (B) abnormal VTA/SN MDD subjects exhibited significantly elevated FA bilaterally in the ventral tegmental area/substantia nigra (VTA/SN), localized to both the SN and the VTA and a trend toward significance in this region in the left hemisphere, while (C) MDD subjects in the normal VTA/SN subgroup did not exhibit any significant FA differences in this region. In contrast, (B) abnormal VTA/SN MDD subjects did not exhibit any significant FA differences overlying the medial forebrain bundle/lateral nucleus of the hypothalamus (MFB/LNH), while (C) normal VTA/SN MDD subjects exhibited a trend toward significant reduction in FA values in this region. Translucent green in images on left indicates *a priori* AOEs for the (B) VTA/SN and (C) MFB/LNH in the slices shown, which were used to constrain the initial contrast analysis. The color bar indicates the range of p values in this figure, from the threshold (p<0.05) to the order of magnitude of the voxel of peak significance (i.e. smallest p value) in the VTA/SN for elevated FA and from the threshold (p<0.05) to the order of magnitude of the voxel of peak significance in the MFB for reduced FA, color in images is viewed using trilinear interpolation. Warm tones (red, orange, yellow) indicate regions in which MDD subjects exhibited elevated FA relative to control subjects; cool tones (blues) indicate regions in which MDD subjects exhibited reduced FA relative to control subjects. RH: right hemisphere; LH: left hemisphere.

A gap in the distribution of mean FA values across MDD subjects was observed for the VTA/SN cluster (Figure 4A). This gap was located at the upper limit of control values; all MDD subjects with values above the gap were outside the range of control values. Gapping analysis indicated it was unlikely that this gap in the MDD VTA/SN FA values would have been observed by chance if this were a homogeneous population (p = 0.001 with a Gaussian distribution; p = 0.005 for a t distribution, df = 4). Ten MDD subjects fell above the gap (“abnormal VTA/SN” subgroup), and 12 fell below it (“normal VTA/SN” subgroup). There were no statistically significant gaps across MDD subjects for the MFB/LNH cluster.

#### b. Voxel-based contrasts of FA maps for the two MDD subgroups

**Figure 5:**
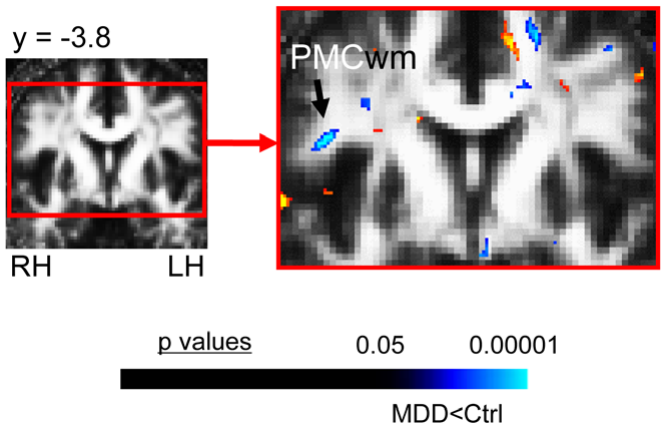
FA difference in the abnormal VTA/SN MDD subgroup in premotor cortex. This figure shows the significant FA difference in MDD subjects versus matched healthy controls for the voxel-based contrast in the abnormal VTA/SN MDD subgroup for AOEs included in our secondary *a priori* hypotheses (10 MDD and 10 controls). In addition to elevated FA in the VTA/SN (Figure 4B), MDD subjects in the abnormal VTA/SN subgroup exhibited significantly reduced FA in white matter adjacent to right premotor cortex (PMCwm). The color bar indicates the range of p values in this figure, from the threshold (p<0.05) to the order of magnitude of the voxel of peak significance (i.e. smallest p value) in PMCwm; color in images is viewed using trilinear interpolation. Cool tones (blues) indicate regions in which MDD subjects exhibited reduced FA relative to control subjects. RH: right hemisphere; LH: left hemisphere.

**Figure 6:**
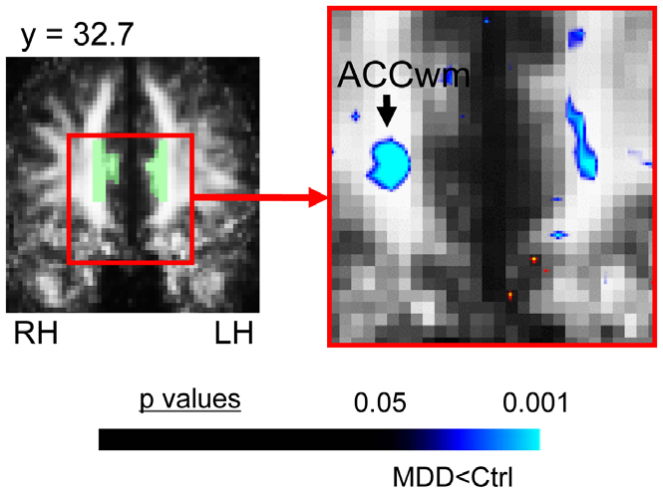
FA difference in the normal VTA/SN MDD subgroup in secondary *a priori* areas of evaluation. This figure shows the FA difference in MDD subjects versus matched healthy controls for the voxel-based contrast in the normal VTA/SN MDD subgroup for AOEs included in our secondary *a priori* hypotheses (12 MMD and 12 controls). In addition to the trend toward reduced FA in the MFB (Figure 4C), MDD subjects in the normal VTA/SN subgroup also exhibited a trend toward reduced FA in white matter adjacent to the right anterior cingulate (ACCwm). Translucent green in image on left indicates the *a priori* AOE for ACCwm in the slices shown, which were used to constrain the initial contrast analysis. The color bar indicates the range of p values in this figure, from the threshold (p<0.05) to the order of magnitude of the voxel of peak significance (i.e. smallest p value) in ACCwm; color in images is viewed using trilinear interpolation. Cool tones (blues) indicate regions in which MDD subjects exhibited reduced FA relative to control subjects. RH: right hemisphere; LH: left hemisphere.

MDD subgroups exhibited two non-overlapping sets of brain microstructural abnormalities when contrasts were conducted separately (Figures 4, 5, 6).

*(1) Abnormal VTA/SN MDD subgroup versus matched control subjects (* *Table 3* *,* *Figures 4* *,* *5* *)*. For MDD subjects with FA values above the gap, a VTA/SN cluster in the right hemisphere covered a significant proportion of the SN and its peak was centered within the SN (Figure 4B). Voxels in this cluster extended into the lateral VTA. There was also a cluster of significantly elevated FA in the left hemisphere VTA/SN (Figure 4B). This subgroup exhibited significantly reduced FA in white matter underlying right premotor cortex (Figure 5). Mean FA values for clusters are reported in Table S4. This subgroup did not exhibit FA abnormalities within the MFB/LNH *a priori* AOE (Figure 4C), or within ACC white matter.

*(2) Normal VTA/SN MDD subgroup versus matched control subjects (* *Table 4* *;* *Figures 4* *,* *6* *)*. The MDD subgroup with VTA/SN FA values below the gap, exhibited a trend toward reduced FA in the left MFB/LNH (Figure 4C), and a trend toward reduced FA in white matter adjacent to right ACC (Figure 6). Mean FA values within clusters are reported in Table S6. This subgroup did not exhibit FA abnormalities within the VTA/SN *a priori* AOE (Figure 4B) or premotor white matter.

#### c. Symptom measures in abnormal versus normal VTA/SN MDD subgroups

**Figure 7:**
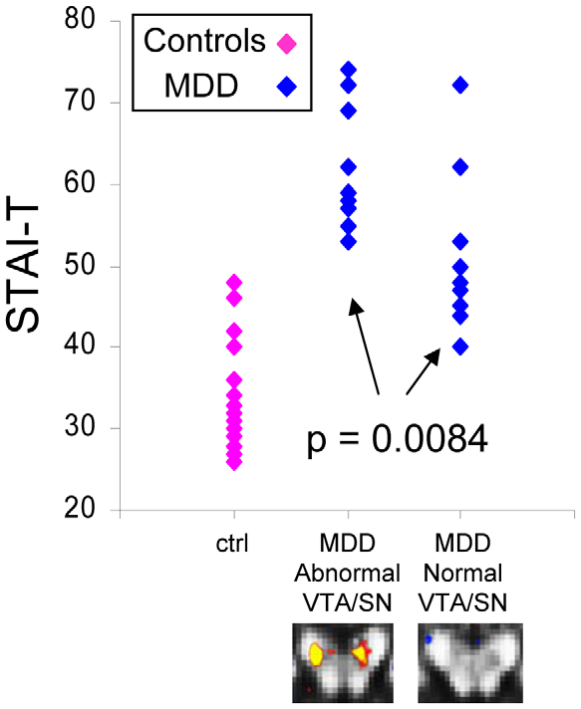
STAI-T (trait anxiety) scores differed between abnormal and normal VTA/SN MDD subgroups. This figure shows the STAI-T scores for MDD subgroups and for control subjects (n = 10 abnormal VTA/SN MDD; n = 11 normal VTA/SN MDD; n = 19 controls). These scores exhibited a similar distribution to VTA/SN FA values (Figure 4A) across MDD subgroups and controls: Scores for MDD subjects in the abnormal VTA/SN subgroup (left column of blue data points) did not overlap with control subject scores (pink data points), whereas the range of scores for subjects in the normal VTA/SN subgroup (right column of blue data points) overlapped with control scores; scores showed a statistically significant difference between MDD subgroups. The control subject score range also extended below the range for MDD subjects in the normal VTA/SN subgroup, as it did for control VTA/SN FA values.

Total IDS-SR scores, and scores for items relating to anhedonia, sadness, and psychomotor symptoms did not differ between the normal and abnormal VTA/SN subgroups (Table 5). In contrast, trait anxiety scores (STAI-T) were significantly different between abnormal and normal VTA/SN MDD subgroups [t(2,19) = 2.96, p = 0.0084, normal VTA/SN mean = 51.27±8.96, abnormal VTA/SN mean = 61.80±7.32; Figure 7].

**Table 5:** MDD Symptom Measures in Abnormal Versus Normal VTA/SN MDD Subgroups.

Symptom	MeanAbnormal VTA/SN subgroup	MeanNormal VTA/SN subgroup	*t test* statistics
Depression severity (IDS-SR)	38.00±10.58	34±8.68	t(2,16) = 1.24, p = 0.23
Anhedonia	1.38±0.52	1.25±0.97	t(2,18) = 0.38, p = 0.71
Sadness	2.44±0.53	1.83±1.03	t(2,19) = 1.77, p = 0.095
Psychomotor symptoms	1.25±0.89	1.00±1.04	t(2,18) = 0.58, p = 0.57
Trait anxiety	61.80±7.32	51.27±8.96	t(2,19) = 2.96, p = 0.0084

## Discussion

In this study, MDD subjects exhibited brain microstructural differences in subcortical reward/aversion regions, specifically the VTA/SN, compared to control subjects. Individual FA values in the VTA/SN divided the MDD cohort into subgroups with distinct profiles of microstructural abnormalities and different levels of trait anxiety, but no difference in other clinical symptoms of MDD. This subtyping supports a hypothesis that etiology and symptoms of MDD may not match one-to-one, although distinguishing clinical factors may be associated with a given etiology.

### Evidence That MDD Is Characterized By Reward/Aversion Circuitry Abnormalities

#### FA abnormalities in the VTA/SN region

In the initial voxel-based contrast, FA was elevated in MDD subjects at the border of the SN and cerebral peduncle in a region that, according to anatomic atlases, contains nigrostriatal projection fibers. Because the initial contrast included a number of patients who did not exhibit the abnormality in the VTA/SN, we viewed the voxel-based contrast for the MDD subgroup with abnormal VTA/SN FA as a better reflection of the location and extent of this abnormality. This follow-up analysis revealed a patient/control difference peak centered within the SN, which covered a significant proportion of the SN and extended into the lateral VTA in both hemispheres, suggesting the VTA/SN finding was not a localized effect specific to the nigrostriatal fibers, and that the effect was seen in the nucleus, rather than the adjacent white matter.

#### Etiological considerations for increased FA in the VTA/SN

The goal of the current study was to identify regions in which brain microstructure was different in MDD, independent of specific etiology, which can be associated with other known structural abnormalities, clinical or behavioral features in a population. Although DTI evaluates water diffusion properties and therefore can be influenced by many potential etiologies, there are certain etiologies commonly associated with abnormalities in white matter, and emerging evidence for abnormalities underlying FA changes in mixed gray/white matter regions; these can be considered here in relation to generating new hypotheses to test in MDD. Specifically, white matter FA is influenced by the integrity of axons and myelin, as well as directional coherence of axons [74], with reduced FA suggesting either a loss of axonal integrity or coherence. Although the basis for altered FA in gray matter regions has not been as well studied, a number of potential alterations in cell or axonal composition within these regions may alter water diffusion properties; this includes reduced cell density and/or cell loss, altered cell structure, or altered integrity or orientation of the cells or axons projecting out of the region. Altered FA has been previously shown in brainstem nuclei in the presence of known neuronal loss, including altered FA in the SN in Parkinson's Disease [e.g. [65], [66]], confirming the potential for FA to detect pathology-relevant changes in the VTA/SN region.

While neuronal loss, as occurs in Parkinson's Disease, would be expected to lead to reduced FA [65], [66], a reduction in glial density could potentially lead to elevated FA due to an increase in the ratio of axons to cell bodies. Evidence for such an effect is seen in a recent study in a mouse model of Pelizaeus-Merzbacher disease (PMD), in which there is transient astrocytic hypertrophy in females. Hypertrophy in these animals was associated with reduced FA, while FA subsequently increased upon the reversal of hypertrophy [75]; i.e. when glial density decreased, FA increased. Elevated FA in a mixed gray/white matter region has also been directly demonstrated in association with induction of a disease process. A recent study showed that an animal model of febrile seizures exhibited elevated FA in the hippocampus following seizure induction [76]. In humans, such seizures promote hyperexcitability of the limbic system and are accompanied by structural and metabolic abnormalities of the limbic system.

Given that reduced glial density has been demonstrated in MDD in the SGC [77] along with imaging abnormalities in this and other prefrontal gray matter regions in MDD, [34], [78], [79], we suggest that future studies can test the hypothesis that there may also be reduced glial density in the VTA/SN in conjunction with the FA abnormality observed here. Since glia are thought to be critical in synapse formation and synaptic plasticity [80], [81], reduced density and/or loss of glia would have a critical impact on information processing in the reward/aversion circuitry. An alternate possibility is that cell morphology is altered in MDD subjects. Russo and colleagues have shown that chronic drug use leads to a reduction in neuronal size in the VTA in an animal model of chronic opiate addiction [82]. This size reduction was accompanied by a reduction in the rewarding effects of morphine. In MDD, analogous factors, such as chronic stress, might have a similar effect on neurons in this region.

#### Functional implications of the VTA/SN abnormality for MDD and reward/aversion processing

Within the VTA/SN region, the VTA predominantly processes reward/aversion information [18], [27], while the SN processes both reward/aversion and motor information [83]. The SN is thought to participate in modulating reward prediction and expectancy [84], as well as prediction of aversive and negatively valenced stimuli [18]. In addition, medial portions of the SN project to and receive projections back from the ventral striatum [30], [70], which processes reward/aversion information [21], while more lateral portions project to the dorsal striatum, which processes motor information [83]. These regions appear to be interconnected in an ascending spiral so that information relating to reward/aversion and motor function is likely mixed in this circuitry [83]. Other efferents of the SN include GABAergic nigro-collicular pathways which have been shown to mediate fear/defense reactions [85].

Taken together, the literature above suggests a hypothesis that altered systems for reward/aversion prediction might lead to an alteration in the capacity of some MDD subjects to assess realistic likelihoods of aversive events [86]. Such individuals, as a consequence, would be likely to show impaired expectancies around negative events and compensate by maintaining higher levels of vigilance/arousal for bad outcomes, which would be clinically observed in the form of higher anxiety [87], [88]. The observation of significantly higher levels of trait anxiety in the abnormal VTA/SN MDD subgroups supports such a hypothesis. Given that the SN and VTA each project to the ventral striatum [89], our findings are also consistent with reports of altered nucleus accumbens function in MDD [2], [32], supporting earlier hypotheses of such an effect [1].

#### FA abnormalities in premotor cortex

Independent of *a priori* regions, MDD subjects exhibited a trend toward reduced FA in white matter underlying premotor cortex in the cohort as a whole, which was significant in the abnormal VTA/SN group. Since a number of tracts run through this region, we cannot be certain that this finding reflects an abnormality in motor fibers. Nevertheless, this finding is of interest in conjunction with the FA abnormality in the SN, given these regions are each key components of motor circuitry, and some MDD subjects in our cohort exhibited psychomotor symptoms.

### Evidence For Subtyping of the MDD Cohort Based on DTI Measures and Relationship to Depressive Symptoms

There are three findings arguing that subjects with normal versus abnormal VTA/SN FA fell into two biologically relevant subtypes. The first is the observed gap in the middle of the distribution of FA values for MDD subjects, which coincided with the cut-off for control values. The second is that there was no overlap in regional localization of FA abnormalities for the two subgroups when evaluated separately. The third is the difference in trait anxiety between groups, with higher mean trait anxiety in the abnormal VTA/SN subgroup. The evidence here for MDD subtyping based on features of the VTA/SN resonates with a recent finding of differences in midbrain resting metabolism between groups of patients whose depressive symptoms did versus did not remit in response to antidepressants [31]. Although Milak and colleagues [31] did not test whether there was a division on an individual basis, their findings suggest the possibility that the mictrostructural subgroups in the current study may also predict treatment responsiveness and could be tested as a potential diagnostic/prognostic biomarker. Future studies will be necessary to evaluate the relationship between altered microstructure and treatment responsiveness.

In contrast, no significant differences in two core symptoms of depression (anhedonia and sadness) or overall depression severity were observed between the two MDD subgroups. While this could potentially reflect a power issue, it is also possible that the neural subgroups observed here are an example of how the same illness can arise when related components of the reward/aversion circuitry are “hit” in different places. These data support the idea that it may be the functional system (i.e. reward/aversion) hit, and not the specific etiology of that hit, that determines whether an individual develops MDD [1], [48], [90]. There is, however, potential for vast differences in a subset of clinical symptoms, such as anxiety, and in treatment responsiveness across different biological etiologies.

The higher trait anxiety levels in the abnormal VTA/SN subgroup are consistent with research connecting the VTA/SN to expectancy processing [84], and connects with the idea that abnormal anxiety may reflect altered expectancy [86], [88]. Elevated trait anxiety in this subgroup is also consistent with findings by Berton & colleagues[25] of VTA abnormalities in an animal model of MDD induced by chronic stress. Clinically, our findings in the VTA/SN support hypotheses regarding involvement of the midbrain dopamine reward circuitry in MDD [7], and support investigation of interventions based thereon [91].

### Limitations of the Interpretation of Our Findings

Several factors inherent to patient imaging studies must be considered for interpretation of findings such as ours. These include the cohort size in relation to subgrouping, the balance between strengths and weaknesses of our selection of registration methods, the issue of whether microstructural abnormalities are primary or secondary to MDD, and medication status of patients. These factors are discussed below.

#### Cohort size, subgrouping, and thresholding

Our search for heterogeneity within the MDD cohort was primarily for the purpose of showing potential reasons previous DTI studies may have exhibited false negatives in subcortical regions. The potential clinical relevance of the subgroups identified is of great interest; however, we emphasize that these findings are preliminary and need to be replicated in larger cohorts. It is also possible that either the cohort size of the subgroups or our conservative thresholds led to false negatives in the current study, particularly in regions that reached the level of a trend (e.g. the MFB). The current study provides justification for future large-scale studies to verify the reproducibility of our findings. Such studies can be conducted using, not only DTI, but also complementary techniques such as post-mortem histological evaluation, which require directed hypotheses such as those generated here.

#### Registration Methods

Because our hypotheses focused on specific components of subcortical reward circuitry, we used registration methods which would maximize precise registration of these regions. It is therefore important to emphasize that alternative registration methods might have more accurately or reliably detected abnormalities in cortical white matter regions in MDD subjects. We did, however, focus some of our manual registration points on ventral and medial prefrontal cortical regions to maximize the likelihood that the SGC, FOC, and ACC regions included in our secondary *a priori* hypotheses would be well registered.

#### Spatial resolution of our a priori regions

Small subcortical nuclei and small white matter fiber tracts inherently have lower effective spatial resolution than cortical regions, and this is a limitation particularly when using the spatial resolution of standard DTI sequences (2 mm). In the current study we aimed to optimize the signal at this spatial resolution by using directed registration methods to maximize alignment in our *a priori* regions. We also note in our Methods that previous studies have successfully detected abnormalities in the VTA/SN region when gross pathology was present [65], [66], suggesting that detection of biological abnormalities in this region should be feasible.

#### Potential confounds of cardiorespiratory movement

Because the brainstem is particularly susceptible to motion artifact from arterial or respiratory pulsation [92], it cannot be ruled out that such movements influenced our data. However, in the case of DTI, image acquisition is integrated over a significant period of time (∼10 minutes), and is therefore less susceptible than functional MRI (fMRI) to the effects of such artifacts leading to individual differences. Unless patients exhibited different cardiorespiratory features than controls (or patients differed across subgroups), differences in cardiorespiratory function would be expected to average out, or in the worst case lead to increased variance of the signal in one cohort, reducing the likelihood of a statistically significant finding. Furthermore, all subjects in this study were thoroughly assessed by review of systems and physical exam by a physician, from which no such physical differences were discerned across groups or subgroups.

#### Are microstructural abnormalities primary or secondary to MDD?

We cannot be certain whether the observed microstructural abnormalities in our MDD subjects were the cause or the result (or both) of the illness or its symptoms. Future studies in larger cohorts and with repeated measures will be necessary to further assess the primacy of abnormalities observed in our study. Such studies will also be needed to evaluate the effects of other factors secondary to MDD, such as tobacco use. Since only three of the 22 MDD subjects in this cohort were current or previous smokers, it is unlikely that our results reflect FA abnormalities secondary to tobacco use. Specifically, this supports the idea that the VTA/SN abnormality could be observed in the absence of tobacco use. Conversely, the control subject who smoked (and had done so for 37 years) exhibited the second lowest VTA/SN FA value (0.24) in the control cohort, indicating that smoking is not sufficient to produce the VTA/SN FA abnormality.

#### Medication status of MDD subjects and DTI findings

Because animal literature in depression [93], [94] and more recently human literature in movement disorders [95] suggests that treatment of symptoms of a disorder may influence brain microstructure, it is important to consider whether some of the observations in our study might have been brought about by medication.

Three factors argue that our main findings were not affected by medication status, although future studies will be required to prospectively evaluate whether antidepressants affect brain microstructure as detected by DTI. First, because our patient population was drawn partially from recently diagnosed (i.e. untreated) patients or patients who did not respond to antidepressant medications in the past; the cohort here included a number of patients who had either never taken or were not currently taking antidepressants (Table 1). Second, similar proportions of patients in the normal and abnormal VTA/SN subgroups were on medications (3/10 in the abnormal VTA/SN subgroup; 4/12 in the normal VTA/SN subgroup) at the time of scanning. Third, individual data points indicate there were subjects in each of the two MDD subgroups who had never taken antidepressants so it is unlikely that the MDD DTI subgrouping, which was based on individual rather than group measures, was an effect of medication or medication differences between subgroups.

### Conclusion

In this study, MDD subjects exhibited brain microstructural abnormalities in the ventral tegmentum, a primary component of the subcortical reward/aversion circuitry. These abnormalities subdivided the cohort into two subgroups which exhibited similar core depressive symptoms, but differences in trait anxiety. These findings add direct support to the hypothesis that alterations in brain reward/aversion circuitry play a role in the etiology of MDD [1].

## Supporting Information

Text S1 Registration and AOE segmentation methods. Describes the anatomical landmarks used in the manual registration step for FA maps, and the procedures used to segment our a priori areas of evaluation (AOEs).
(0.05 MB DOC)

Figure S1 Registration comparison across groups. Examples of (A) coronal and (B) axial slices through average FA maps for control subjects (left column) and MDD subjects (right column) show that registration accuracy of images used in group contrasts was similar between groups and, to illustrate the visibility of anatomy on these images. Coronal images are at y = -18.3 and axial images at z = 3.5 in MNI Talairach coordinates. RH: right hemisphere; LH: left hemisphere.
(0.91 MB TIF)

Figure S2 Ventral tegmentum area of evaluation (AOE). This figure shows the primary a priori AOE in the VTA/SN. A priori AOEs were segmented on the average control FA map and then used to constrain the voxels considered in the initial contrast analysis for MDD versus control cohorts. The image on the left in (A) illustrates the visibility of regions and landmarks used for segmentation of the VTA/SN and the three images to the right depict the AOE in translucent green from (left to right) an axial view (the primary orientation in which segmentation was done), a coronal view, and a sagittal view. In (B) the AOE is superimposed on the average MDD FA map at the same locations. RH: right hemisphere; LH: left hemisphere.
(0.52 MB TIF)

Figure S3 Medial forebrain bundle area of evaluation (AOE). This figure shows the primary a priori AOE in the medial forebrain bundle (MFB), which also coincided with and included the lateral nucleus of the hypothalamus (LNH). A priori AOEs were segmented on the average control FA map and then used to constrain the voxels considered in the initial contrast analysis for MDD versus control cohorts. The image on the left in (A) illustrates the visibility of regions and landmarks used for segmentation of the MFB/LNH and the three images to the right depict the AOE in translucent green from (left to right) a coronal view (the primary orientation in which segmentation was done), an axial view, and a sagittal view. In (B) the AOE is superimposed on the average MDD FA map at the same locations. RH: right hemisphere; LH: left hemisphere.
(0.65 MB TIF)

Figure S4 Amygdalofugal pathway area of evaluation (AOE). This figure shows the primary a priori AOE in the amygdalofugal pathway, which also coincided with and included the substantia inominota. A priori AOEs were segmented on the average control FA map and then used to constrain the voxels considered in the initial contrast analysis for MDD versus control cohorts. The image on the left in (A) illustrates the visibility of regions and landmarks used for segmentation of the amygdalofugal pathway/substantia inominota and the three images to the right depict the AOE in translucent green from (left to right) a coronal view (the primary orientation in which segmentation was done), an axial view, and a sagittal view. In (B) the AOE is superimposed on the average MDD FA map at the same locations. RH: right hemisphere; LH: left hemisphere.
(0.70 MB TIF)

Figure S5 Ventral tegmentum group difference cluster. This figure shows the cluster detected within the VTA/SN a priori AOE in the voxel-based contrast analysis of MDD versus control cohorts. This cluster was used to extract values from individual FA maps for use in the follow-up analyses (main text) and permutation test (Dataset SI). (A) Images illustrating the average control FA map (left image), and a priori AOE segmented on that map (right image) that was used to constrain the voxel-based analysis. (B) The cluster of voxels (at right, shown on average control and MDD FA maps, respectively) that met the uncorrected p<0.05 cluster threshold on the p map of the initial MDD versus control voxel-based contrast analysis (left image) and fell within the VTA/SN AOE. Clusters were identified on p maps that had not been smoothed, and thus, p maps are illustrated here without smoothing or interpolation. RH: right hemisphere; LH: left hemisphere.
(0.46 MB TIF)

Figure S6 Medial forebrain bundle group difference cluster. This figure shows the cluster detected within the MFB/LNH a priori AOE in the voxel-based contrast analysis of MDD versus control cohorts. This cluster was used to extract values from individual FA maps for use in the follow-up analyses (main text) and permutation test (Dataset SI). (A) Images illustrating the average control FA map (left image), and a priori AOE segmented on that map (right image) that was used to constrain the voxel-based analysis. (B) The cluster of voxels (at right, shown on average control and MDD FA maps, respectively) that met the uncorrected p<0.05 cluster threshold on the p map of the initial MDD versus control voxel-based contrast analysis (left image) and fell within the MFB/LNH AOE. Clusters were identified on p maps that had not been smoothed, and thus, p maps are illustrated here without smoothing or interpolation. RH: right hemisphere; LH: left hemisphere.
(0.51 MB TIF)

Figure S7 Examples of secondary a priori areas of evaluation (AOEs). This figure shows slices through each of the five segmentations included in our secondary a priori AOEs, including white matter adjacent to (A) dorsolateral prefrontal cortex (DLPFCwm), (B) anterior cingulate cortex (ACCwm), (C) paracingulate cortex (PACwm), (D) orbitofrontal cortex (FOCwm), and (E) subgenual prefrontal cortex (SGCwm).
(0.74 MB TIF)

Dataset S1 Permutation tests. Reports the methods and results of the permutation analysis conducted as a complement to the voxel-wise contrasts of FA maps.
(0.12 MB DOC)

Dataset S2 VT abnormalities and laterality. Analysis evaluating whether right hemisphere VT abnormalities in the MDD cohort reflected a loss of normal hemispheric asymmetry in this region or a bilateral change that was simply more significant in one hemisphere than the other.
(0.03 MB DOC)
