# Supplementary material for: Microstructural Abnormalities in Subcortical Reward Circuitry of Subjects with Major Depressive Disorder
Source: PLoS One. 2010 Nov 29;5(11):e13945. doi: 10.1371/journal.pone.0013945 (PMC2993928; doi:10.1371/journal.pone.0013945)
Supplement: Text S1 — Registration and AOE segmentation methods. Describes the anatomical landmarks used in the manual registration step for FA maps, and the procedures used to segment our a priori areas of evaluation (AOEs). (0.05 MB DOC) [file pone.0013945.s001.doc]

**Supporting Information Text I (Text SI): Registration and AOE segmentation methods**

**A. Registration: Landmarks Used in the Manual Registration Step**

Landmarks for the manual registration step of FA maps to the MNI T2 template included: (1) the center of mass of the ventral tegmental area/substantia nigra (VTA/SN) and the boundaries of the midbrain in axial sections running through the VTA/SN (2) the boundaries of the lentiform nucleus in coronal and axial orientations (for registration of ventral subcortical regions), and (3) the outer boundaries of the medial and ventral prefrontal cortical regions. Landmarks were defined by a neuroanatomist (N.M.) For comparison of registration consistency between MDD versus control cohorts, Supporting Information Figure 1 (Figure S1), shows a comparison of average FA maps for the two cohorts after automated and manual registration steps were completed.

**B. Segmentation of *a priori* Areas of Interest (AOEs)**

AOEs were segmented by an anatomist (N.M.) with guidance from landmarks in neuroanatomic atlases [1,2,3] and localization of anatomical structures, which were visible on this average map. Segmentations of these AOEs were done directly on the average FA map for the healthy control group (registered in ICBM152 T2 template space) (1) to ensure that segmentations were exactly registered with the maps being considered and (2) because the signal intensity contrast in the FA maps was better for delineating the subcortical anatomy than the T2 maps acquired as part of the DTI sequence.

**1. Segmentation of a priori AOEs for primary a priori hypothesis.** (1) The VTA/SN AOE was defined within the midbrain. This AOE included voxels of the rostral midbrain caudal to the cerebral peduncles, corresponding to the anatomical location of the SN as well as of the corticonigral, pallidonigral and nigrostriatal fiber systems. It extended to the midline within the tegmentum to include voxels representing the VTA as well. (2) The MFB AOE included voxels within a region defined by the coronal planes at the mammillary bodies caudally and the anterior commissure rostrally, laterally by the internal capsule or the globus pallidus and medially by the lateral hypothalamic gray border extending to the ventral surface of the basal forebrain. (3) Voxels representing the amygdalofugal pathway AOE were defined in a location dorsal to the amygdala within the substantia innominata at coronal sections extending from the anterior commissure to the nucleus accumbens septi. These voxels remained generally lateral to the third ventricle and medial to the lenticular nucleus and, more rostrally, medial to the claustrum. Examples of these AOEs are shown in Figures S2-S4.

These AOEs were used to constrain the regions of the FA map considered (Figures S5,S6) in the voxel-based contrast for the primary set of hypotheses. Individual subject FA values within these clusters (i.e., mean FA across the cluster) were also used for follow-up gapping analyses and correlations with symptom profiles, as well as in the permutation test (Dataset SI) comparing MDD and control cohorts.

**2.** **Segmentation of a priori AOEs for secondary a priori hypotheses.** (1) The DLPFC_WM_ AOE included white matter voxels within the superior and middle frontal gyri on the lateral hemispheric surface [4]. (2) The FOC_WM_ AOE included white matter voxels within the orbital frontal region as defined by Caviness [4]. (3) The ACC_WM_ and PAC_WM_ AOEs included white matter voxels within the pregenual portions of the ACC and PAC gyri respectively. (4) The SGC_WM_ AOE included white matter voxels within the paraterminal gyrus ventral to the corpus callosum [4]. Examples of these AOEs are shown in Figure S7.

These AOEs were used to constrain the regions of the FA map considered in the voxel-based contrast for the secondary set of hypotheses. Individual subject FA values within these clusters (i.e., mean FA across the cluster) were also used in the permutation test comparing MDD and control cohorts (Dataset SI).

**References**

1. Haines D (1991) Neuroanatomy: An atlas of structures, sections and systems.: Williams & Wilkins.

2. Nieuwenhuys R, Voogd J, van Huijzen C (1988) The Human Central Nervous System: A synopsis and atlas. Berlin: Springer-Verlag.

3. Mai JK, Assheuer J, Paxinos G (1997) Atlas of the Human Brain. New York: Academic Press.

4. Caviness VS, Jr., Meyer JW, Makris N, Kennedy DN (1996) MRI-based parcellation of human neocortex: an anatomically specified method with estimate of reliability. Journal of Cognitive Neuroscience 8: 566-588.

**Figure Legends for Text SI**

**Figure S1. Registration comparison across groups.** Examples of (A) coronal and (B) axial slices through average FA maps for control subjects (left column) and MDD subjects (right column) show that registration accuracy of images used in group contrasts was similar between groups and, to illustrate the visibility of anatomy on these images. Coronal images are at y = -18.3 and axial images at z = 3.5 in MNI Talairach coordinates. RH: right hemisphere; LH: left hemisphere.

**Figure S2. Ventral tegmentum area of evaluation (AOE).** This figure shows the primary *a priori* AOE in the VTA/SN. *A priori* AOEs were segmented on the average control FA map and then used to constrain the voxels considered in the initial contrast analysis for MDD versus control cohorts. The image on the left in (A) illustrates the visibility of regions and landmarks used for segmentation of the VTA/SN and the three images to the right depict the AOE in translucent green from (left to right) an axial view (the primary orientation in which segmentation was done), a coronal view, and a sagittal view. In (B) the AOE is superimposed on the average MDD FA map at the same locations. RH: right hemisphere; LH: left hemisphere.

**Figure S3. Medial forebrain bundle area of evaluation (AOE).** This figure shows the primary *a priori* AOE in the medial forebrain bundle (MFB), which also coincided with and included the lateral nucleus of the hypothalamus (LNH). *A priori* AOEs were segmented on the average control FA map and then used to constrain the voxels considered in the initial contrast analysis for MDD versus control cohorts. The image on the left in (A) illustrates the visibility of regions and landmarks used for segmentation of the MFB/LNH and the three images to the right depict the AOE in translucent green from (left to right) a coronal view (the primary orientation in which segmentation was done), an axial view, and a sagittal view. In (B) the AOE is superimposed on the average MDD FA map at the same locations. RH: right hemisphere; LH: left hemisphere.

**Figure S4. Amygdalofugal pathway area of evaluation (AOE).** This figure shows the primary *a priori* AOE in the amygdalofugal pathway, which also coincided with and included the substantia inominota. *A priori* AOEs were segmented on the average control FA map and then used to constrain the voxels considered in the initial contrast analysis for MDD versus control cohorts. The image on the left in (A) illustrates the visibility of regions and landmarks used for segmentation of the amygdalofugal pathway/substantia inominota and the three images to the right depict the AOE in translucent green from (left to right) a coronal view (the primary orientation in which segmentation was done), an axial view, and a sagittal view. In (B) the AOE is superimposed on the average MDD FA map at the same locations. RH: right hemisphere; LH: left hemisphere.

**Figure S5. Ventral tegmentum group difference cluster.** This figure shows the cluster detected within the VTA/SN *a priori* AOE in the voxel-based contrast analysis of MDD versus control cohorts. This cluster was used to extract values from individual FA maps for use in the follow-up analyses (main text) and permutation test (Dataset SI). (A) Images illustrating the average control FA map (left image), and *a priori* AOE segmented on that map (right image) that was used to constrain the voxel-based analysis. (B) The cluster of voxels (at right, shown on average control and MDD FA maps, respectively) that met the uncorrected p < 0.05 cluster threshold on the p map of the initial MDD versus control voxel-based contrast analysis (left image) and fell within the VTA/SN AOE. Clusters were identified on p maps that had not been smoothed, and thus, p maps are illustrated here without smoothing or interpolation. RH: right hemisphere; LH: left hemisphere.

**Figure S6. Medial forebrain bundle group difference cluster.** This figure shows the cluster detected within the MFB/LNH *a priori* AOE in the voxel-based contrast analysis of MDD versus control cohorts. This cluster was used to extract values from individual FA maps for use in the follow-up analyses (main text) and permutation test (Dataset SI). (A) Images illustrating the average control FA map (left image), and *a priori* AOE segmented on that map (right image) that was used to constrain the voxel-based analysis. (B) The cluster of voxels (at right, shown on average control and MDD FA maps, respectively) that met the uncorrected p < 0.05 cluster threshold on the p map of the initial MDD versus control voxel-based contrast analysis (left image) and fell within the MFB/LNH AOE. Clusters were identified on p maps that had not been smoothed, and thus, p maps are illustrated here without smoothing or interpolation. RH: right hemisphere; LH: left hemisphere.

**Figure S7. Examples of secondary a priori areas of evaluation (AOEs).** This figure shows slices through each of the five segmentations included in our secondary a priori AOEs, including white matter adjacent to (A) dorsolateral prefrontal cortex (DLPFCwm), (B) anterior cingulate cortex (ACCwm), (C) paracingulate cortex (PACwm), (D) orbitofrontal cortex (FOCwm), and (E) subgenual prefrontal cortex (SGCwm).
