# Supplementary figures and images for: Microstructural Abnormalities in Subcortical Reward Circuitry of Subjects with Major Depressive Disorder
Source: PLoS One. 2010 Nov 29;5(11):e13945. doi: 10.1371/journal.pone.0013945 (PMC2993928; doi:10.1371/journal.pone.0013945)

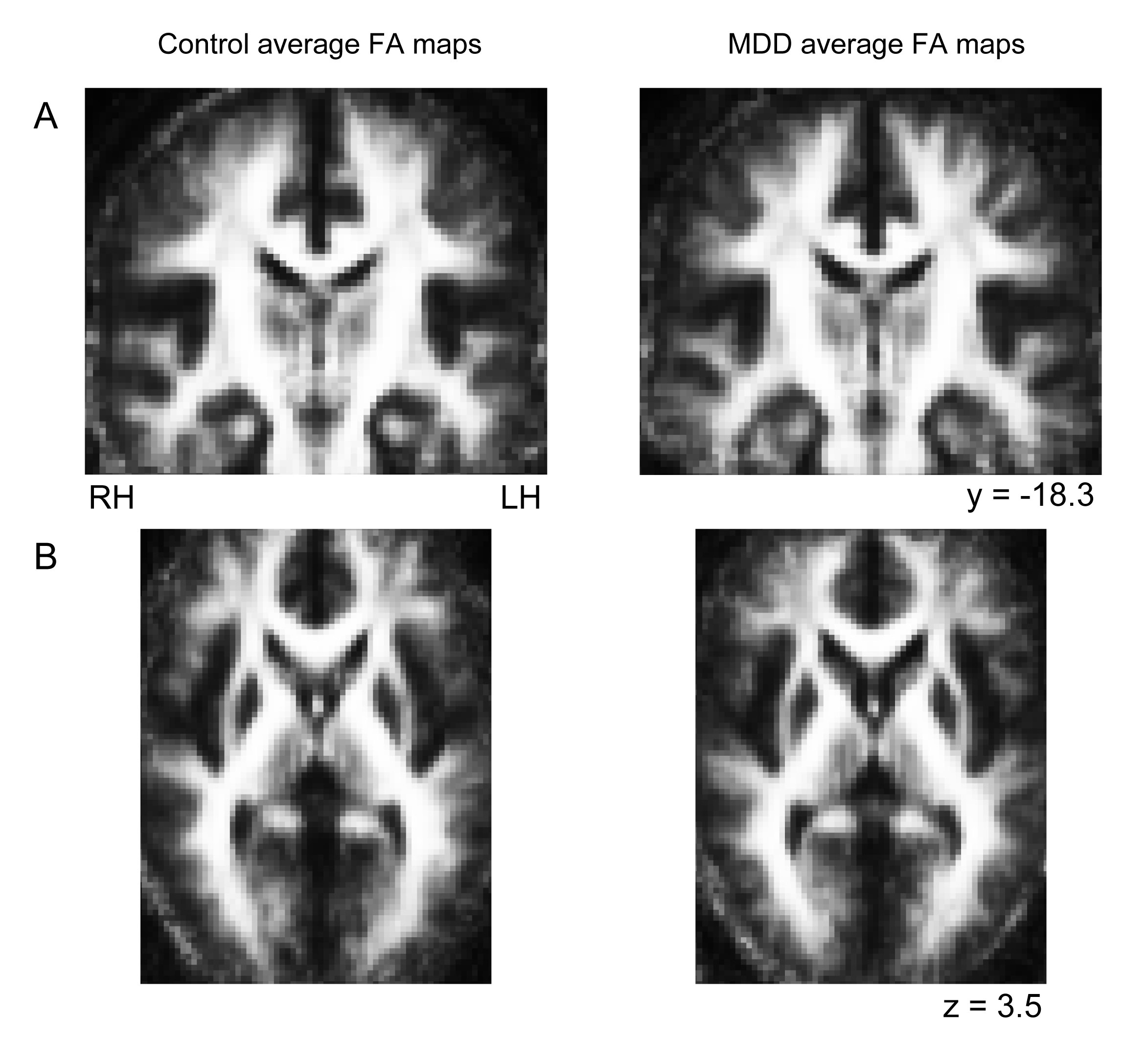

Supplement: Figure S1 — Registration comparison across groups. Examples of (A) coronal and (B) axial slices through average FA maps for control subjects (left column) and MDD subjects (right column) show that registration accuracy of images used in group contrasts was similar between groups and, to illustrate the visibility of anatomy on these images. Coronal images are at y = -18.3 and axial images at z = 3.5 in MNI Talairach coordinates. RH: right hemisphere; LH: left hemisphere. (0.91 MB TIF) [file pone.0013945.s002.tif]

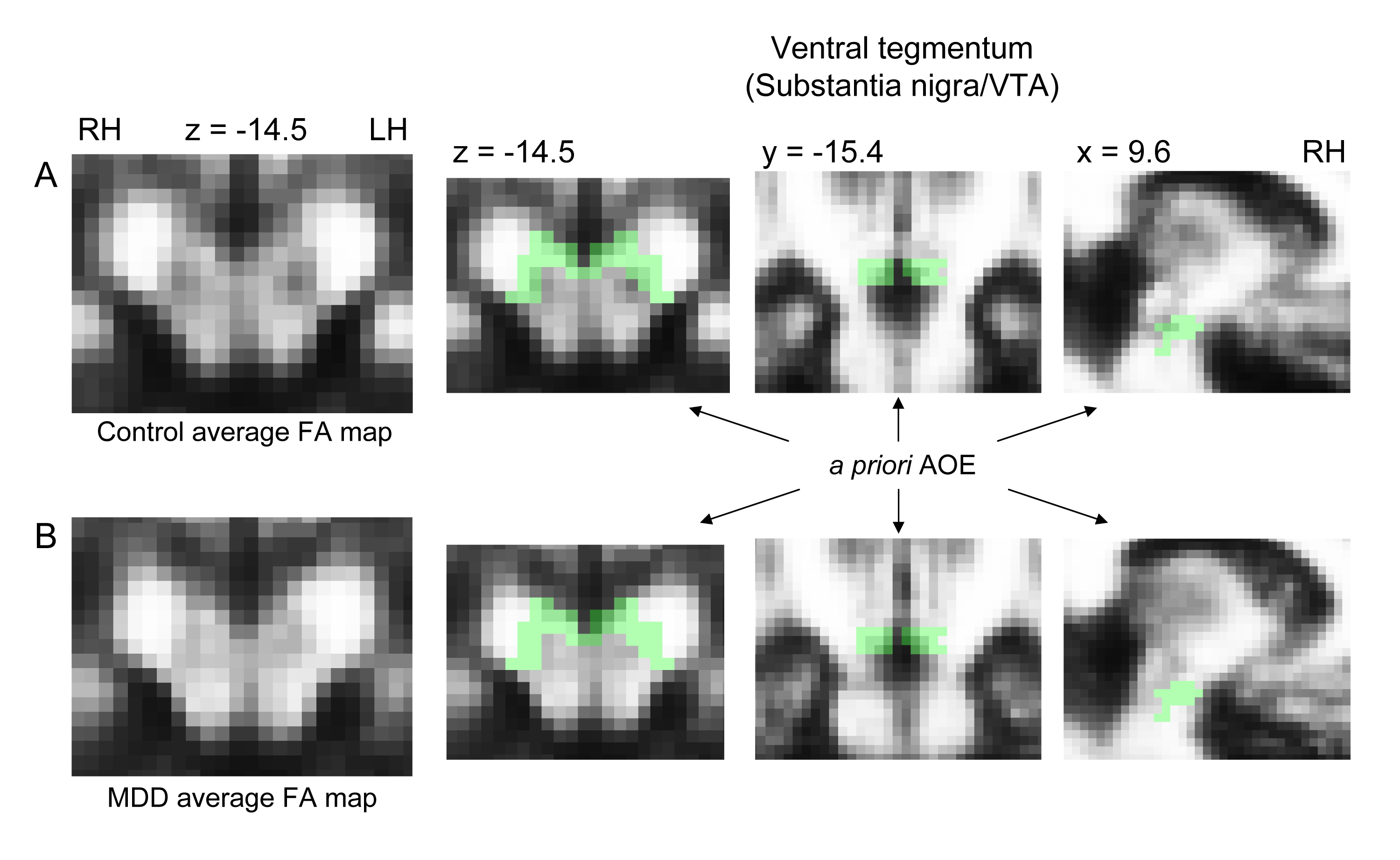

Supplement: Figure S2 — Ventral tegmentum area of evaluation (AOE). This figure shows the primary a priori AOE in the VTA/SN. A priori AOEs were segmented on the average control FA map and then used to constrain the voxels considered in the initial contrast analysis for MDD versus control cohorts. The image on the left in (A) illustrates the visibility of regions and landmarks used for segmentation of the VTA/SN and the three images to the right depict the AOE in translucent green from (left to right) an axial view (the primary orientation in which segmentation was done), a coronal view, and a sagittal view. In (B) the AOE is superimposed on the average MDD FA map at the same locations. RH: right hemisphere; LH: left hemisphere. (0.52 MB TIF) [file pone.0013945.s003.tif]

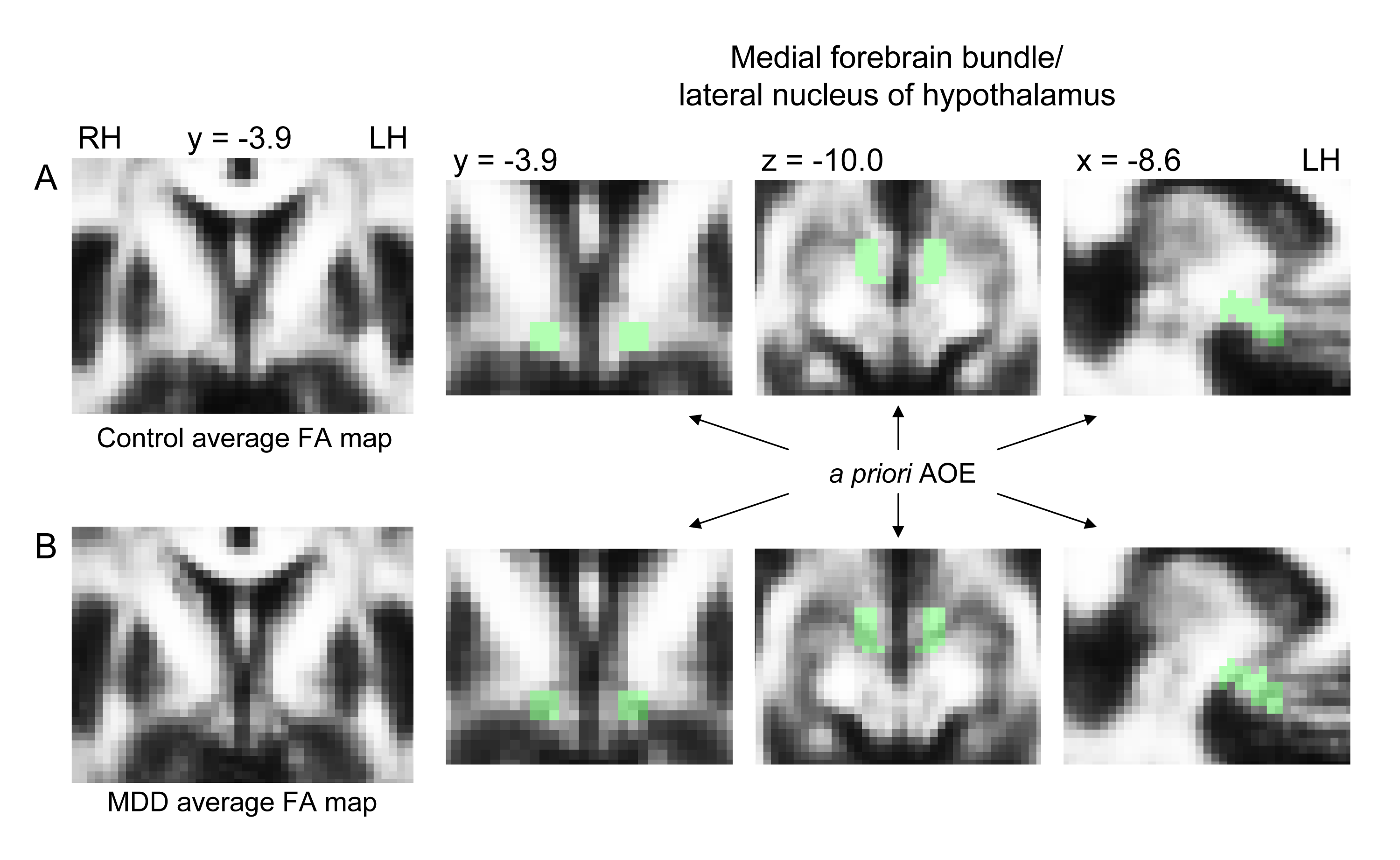

Supplement: Figure S3 — Medial forebrain bundle area of evaluation (AOE). This figure shows the primary a priori AOE in the medial forebrain bundle (MFB), which also coincided with and included the lateral nucleus of the hypothalamus (LNH). A priori AOEs were segmented on the average control FA map and then used to constrain the voxels considered in the initial contrast analysis for MDD versus control cohorts. The image on the left in (A) illustrates the visibility of regions and landmarks used for segmentation of the MFB/LNH and the three images to the right depict the AOE in translucent green from (left to right) a coronal view (the primary orientation in which segmentation was done), an axial view, and a sagittal view. In (B) the AOE is superimposed on the average MDD FA map at the same locations. RH: right hemisphere; LH: left hemisphere. (0.65 MB TIF) [file pone.0013945.s004.tif]

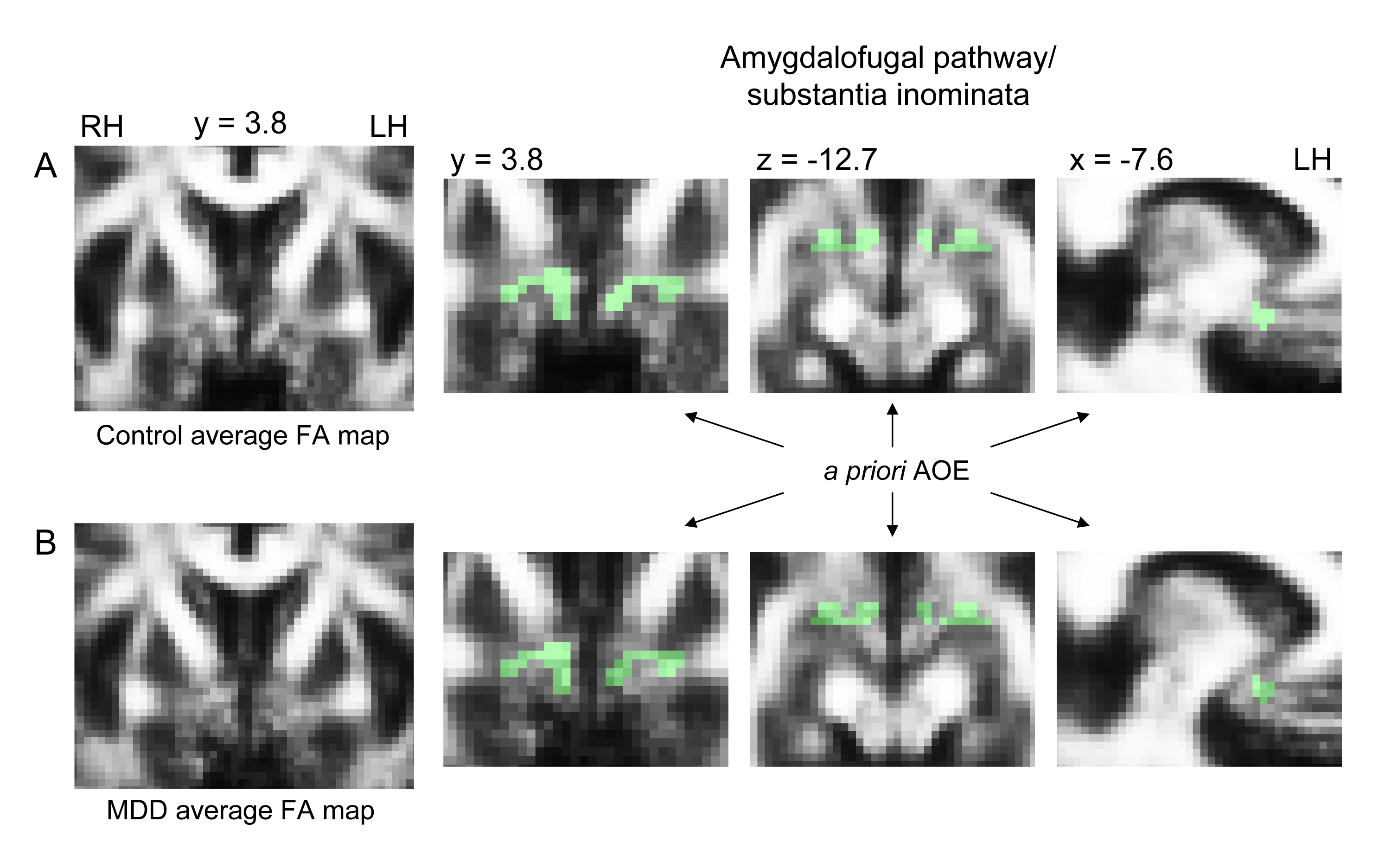

Supplement: Figure S4 — Amygdalofugal pathway area of evaluation (AOE). This figure shows the primary a priori AOE in the amygdalofugal pathway, which also coincided with and included the substantia inominota. A priori AOEs were segmented on the average control FA map and then used to constrain the voxels considered in the initial contrast analysis for MDD versus control cohorts. The image on the left in (A) illustrates the visibility of regions and landmarks used for segmentation of the amygdalofugal pathway/substantia inominota and the three images to the right depict the AOE in translucent green from (left to right) a coronal view (the primary orientation in which segmentation was done), an axial view, and a sagittal view. In (B) the AOE is superimposed on the average MDD FA map at the same locations. RH: right hemisphere; LH: left hemisphere. (0.70 MB TIF) [file pone.0013945.s005.tif]

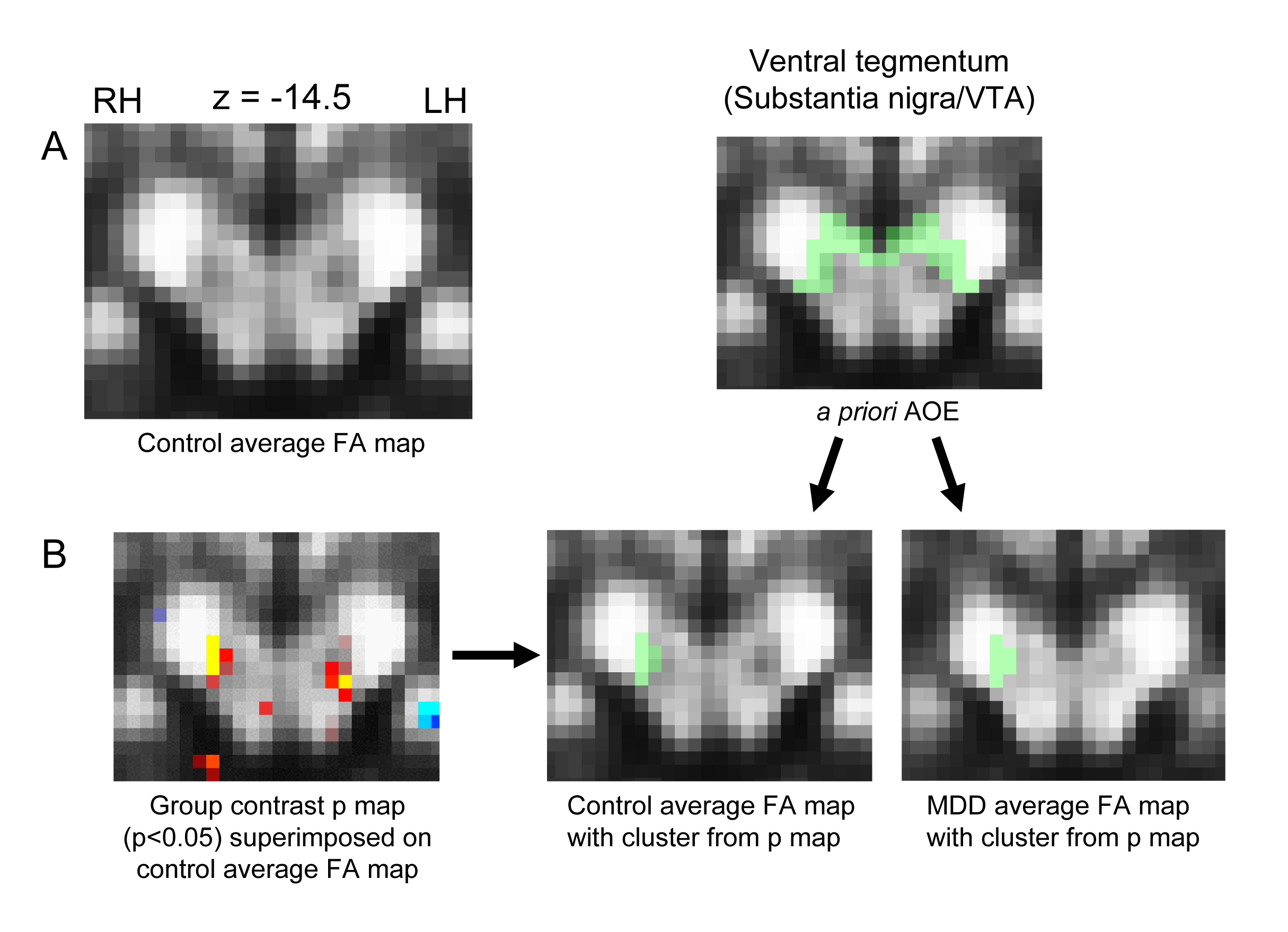

Supplement: Figure S5 — Ventral tegmentum group difference cluster. This figure shows the cluster detected within the VTA/SN a priori AOE in the voxel-based contrast analysis of MDD versus control cohorts. This cluster was used to extract values from individual FA maps for use in the follow-up analyses (main text) and permutation test (Dataset SI). (A) Images illustrating the average control FA map (left image), and a priori AOE segmented on that map (right image) that was used to constrain the voxel-based analysis. (B) The cluster of voxels (at right, shown on average control and MDD FA maps, respectively) that met the uncorrected p<0.05 cluster threshold on the p map of the initial MDD versus control voxel-based contrast analysis (left image) and fell within the VTA/SN AOE. Clusters were identified on p maps that had not been smoothed, and thus, p maps are illustrated here without smoothing or interpolation. RH: right hemisphere; LH: left hemisphere. (0.46 MB TIF) [file pone.0013945.s006.tif]

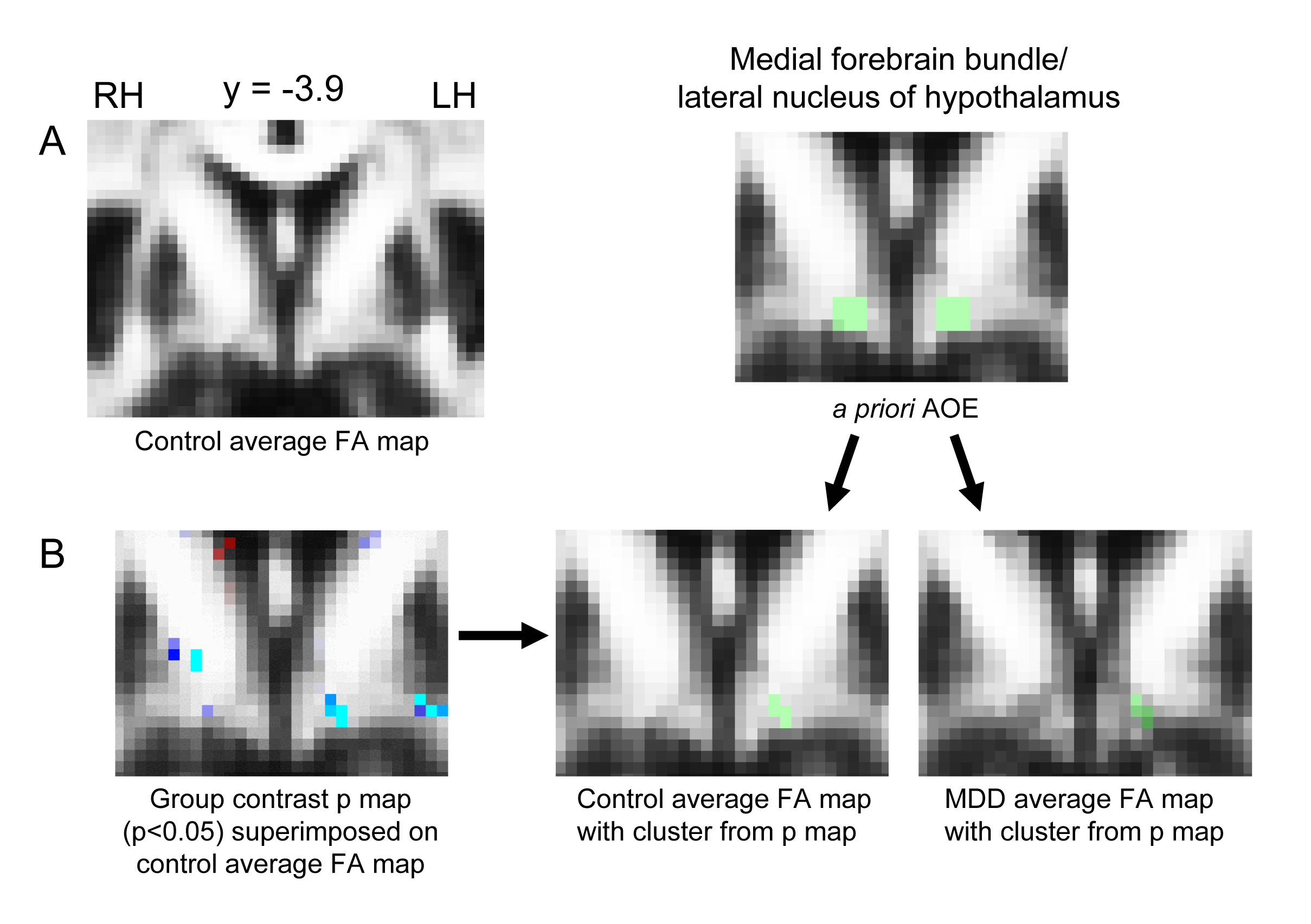

Supplement: Figure S6 — Medial forebrain bundle group difference cluster. This figure shows the cluster detected within the MFB/LNH a priori AOE in the voxel-based contrast analysis of MDD versus control cohorts. This cluster was used to extract values from individual FA maps for use in the follow-up analyses (main text) and permutation test (Dataset SI). (A) Images illustrating the average control FA map (left image), and a priori AOE segmented on that map (right image) that was used to constrain the voxel-based analysis. (B) The cluster of voxels (at right, shown on average control and MDD FA maps, respectively) that met the uncorrected p<0.05 cluster threshold on the p map of the initial MDD versus control voxel-based contrast analysis (left image) and fell within the MFB/LNH AOE. Clusters were identified on p maps that had not been smoothed, and thus, p maps are illustrated here without smoothing or interpolation. RH: right hemisphere; LH: left hemisphere. (0.51 MB TIF) [file pone.0013945.s007.tif]

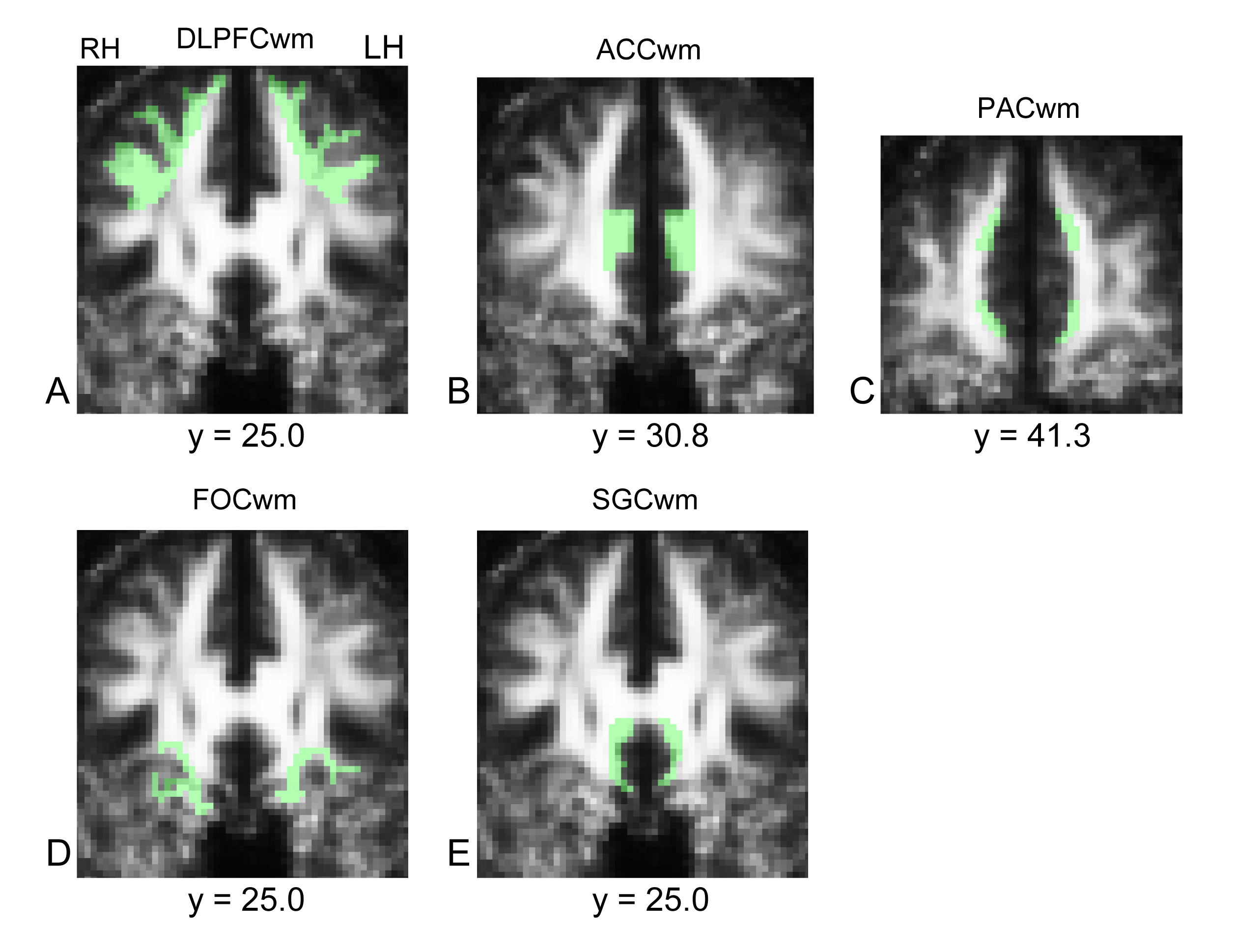

Supplement: Figure S7 — Examples of secondary a priori areas of evaluation (AOEs). This figure shows slices through each of the five segmentations included in our secondary a priori AOEs, including white matter adjacent to (A) dorsolateral prefrontal cortex (DLPFCwm), (B) anterior cingulate cortex (ACCwm), (C) paracingulate cortex (PACwm), (D) orbitofrontal cortex (FOCwm), and (E) subgenual prefrontal cortex (SGCwm). (0.74 MB TIF) [file pone.0013945.s008.tif]
