# Supplementary material for: Microstructural Abnormalities in Subcortical Reward Circuitry of Subjects with Major Depressive Disorder
Source: PLoS One. 2010 Nov 29;5(11):e13945. doi: 10.1371/journal.pone.0013945 (PMC2993928; doi:10.1371/journal.pone.0013945)
Supplement: Dataset S1 — Permutation tests. Reports the methods and results of the permutation analysis conducted as a complement to the voxel-wise contrasts of FA maps. (0.12 MB DOC) [file pone.0013945.s009.doc]

**Supporting Information Dataset I (Dataset SI): Permutation tests**

**A. Methods**

**1. Permutation test for main contrast analysis..**As a supplemental analysis, mean FA values across clusters were used to perform a permutation test comparing MDD and control cohorts. The permutation test provided additional evidence that group differences were unlikely to be false positives due to registration or anatomy differences unrelated to diagnosis. Specifically, the mean FA across each cluster identified in the voxel-based contrast analysis was calculated for each subject using their individual, registered FA map. The test was then done by (1) computing the t-statistic (using a two-tailed test) for observed MDD/control group differences in mean FA for each cluster; (2) randomly assigning subjects to groups without replacement while maintaining the observed group sample sizes; (3) computing the absolute t-value for this random assignment of subjects to groups; (4) repeating steps (2) and (3) for all possible permutations, or a maximum of 10^6 random balanced permutations; (5) computing the proportion of permutation tests that resulted in a t-statistic greater than or equal to the absolute value of the observed t-statistic in (1).

**2. Permutation test for follow-up analyses**. Clusters were identified in these voxel-based contrasts using the same cluster thresholds as in the initial group contrast, and mean FA from clusters was subjected to a permutation test and correction for multiple comparisons using the same methods as described for the initial group contrast.

**B. Results**

**1. Permutation test for main contrast analysis.**

*a. Primary a priori hypothesized areas of evaluation (AOEs) (Supporting Information Table 1 or Table S1; Figure 1).* Within our primary *a priori* AOEs, the voxel-based contrast analysis (i.e. voxel-by-voxel comparisons) and permutation test detected significantly elevated FA in MDD subjects within the VTA/SN. Specifically, FA was elevated at the ventral/lateral edge of the right SN adjacent to the cerebral peduncle and overlying the nigral fiber system (striatonigral, nigrostriatal, and corticostriatal fibers; Figure 1). There was also a trend toward reduced FA in the MDD cohort in the left MFB where it passes through the lateral nucleus of the hypothalamus (LNH). MDD subjects did not exhibit significant FA differences from controls in the *a priori* AOE overlying the amygdalofugal pathway. Mean FA values for each cluster detected in the voxel-based contrast are reported in Table S2.

In contrast to the right VTA/SN findings, only three contiguous voxels at p<0.05 were observed within the left VTA/SN in the initial group contrast (i.e. this was below the cluster threshold). Despite this apparent laterality of the VTA/SN finding, a permutation test comparing values in the right VTA/SN cluster against values in a mirror image cluster in the left hemisphere indicated that FA in the VTA/SN region did not differ significantly across the two hemispheres in the MDD cohort (p=0.45). The control cohort showed a trend toward a hemispheric difference in this region, with lower FA in the left than the right VTA/SN (p=0.065).

*b. Secondary a priori hypothesized AOEs (Table S1; Figure 2).* Within our secondary *a priori* AOEs [i.e., orbitofrontal cortex (FOC), anterior cingulate cortex (ACC), paracingulate cortex (PAC), subgenual prefrontal cortex (SGC), and dorsolateral prefrontal cortex (DLPFC)], the voxel-based contrast analysis and permutation test detected significantly reduced FA in white matter adjacent to two white matter regions underlying DLPFC (Fig 2). There were also trends toward significance in the right ACC and left ACC/PAC. Mean FA values for each cluster detected in the voxel-based contrast are reported in Table S2.

*c. Other regions meeting the cluster threshold (Table S1; Figure 3).* There were two regions outside the *a priori* AOEs that reached the cluster threshold of 81 contiguous voxels, at p<0.05 per voxel, and significance in the permutation test. These included reduced FA in white matter localized within the right precentral gyrus, specifically below the premotor cortex (BA6) according to Talairach referencing (Figure 3A). MDD subjects also exhibited elevated FA in white matter adjacent to right calcarine cortex (Figure 3B). A cluster in white matter underlying left premotor cortex exhibited a trend toward significance. Mean FA values for each cluster detected in the voxel-based contrast are reported in Table S2.

**2. Permutation test for follow-up analyses.**

*a. Voxel-based contrasts of FA maps for the two MDD subgroups.* Because the gapping analysis suggested there were two “subgroups” of MDD subjects, we recalculated the voxel-based contrasts separately for each of these subgroups versus their individually matched controls to further evaluate whether these might represent two distinct neural populations. The two MDD subgroups exhibited two non-overlapping sets of brain microstructural abnormalities in these contrasts (Figures 4-6).

*b. Abnormal VTA/SN MDD subgroup versus matched control subjects (Table S3, Figures 4,5).* The MDD subgroup that exhibited VTA/SN FA values outside the range of controls (i.e. above the gap), exhibited elevated FA across broad regions of the VTA/SN bilaterally in the follow-up contrast analysis and permutation test, and thus will be referred to as the “abnormal VTA/SN” MDD subgroup. The cluster of elevated FA in the right hemisphere VTA/SN covered a significant proportion of the SN and the peak was centered within the SN (Figure 4B). The voxels in this cluster also extended into the lateral VTA. There was a trend toward elevated FA in the left hemisphere VTA/SN in these subjects. The left hemisphere cluster fell more predominantly within the lateral VTA than the SN, although a substantial portion of the cluster in this hemisphere was posteromedial to the SN and VTA, with the anterolateral edge overlapping the SN (Figure 4B). There were no hemispheric differences in VTA/SN FA in either the MDD or the control cohorts included in the abnormal VTA/SN subgroup contrast (MDD: p=0.66 for mirror image of right hemisphere cluster, p=0.34 for statistically defined cluster; control: p=0.26 for mirror image of right hemisphere cluster, p=0.34 for statistically defined cluster). The abnormal VTA/SN MDD subgroup did not exhibit significant FA abnormalities within the MFB/LNH *a priori* AOE (Figure 4C), or within ACC or PAC white matter.

The analysis of FA within the abnormal VTA/SN subgroup also showed a trend toward reduced FA in left DLPFC and a trend toward elevated FA in FOC gray matter (Table S3, Figure 5). This subgroup also exhibited a trend toward reduced FA in white matter underlying right premotor cortex.

Mean FA values for each cluster detected in the voxel-based contrast for this subgroup are reported for each cohort in Table S4.

*c. Normal VTA/SN MDD subgroup versus matched control subjects (Table S5; Figures 4,6).* The MDD subgroup exhibiting VTA/SN FA values within the range of controls (i.e. below the gap), exhibited significantly reduced FA in white matter adjacent to right ACC (Figure 6) and a trend toward reduced FA in the left MFB/LNH (Figure 4C) in the follow-up contrast analysis and permutation test. This MDD subgroup did not exhibit significant FA abnormalities within the VTA/SN *a priori* AOE (Figure 4B), DLPFC white matter, prefrontal gray matter, or premotor white matter. This group will thus be referred to as the “normal VTA/SN” MDD subgroup.

Mean FA values for each cluster detected in the voxel-based contrast for this subgroup are reported in Table S6.

**Tables for Dataset SI**

| **Table S1. Permutation Test for the Whole Cohort (22 MDD Versus 22 Matched Controls)** | | | | | |
| --- | --- | --- | --- | --- | --- |
| **Region** | **MNI coordinates at peak difference** | | **Cluster size**  **(# voxels)** | | **p value in permutation test** |
| **FA group differences which met the 9-voxel cluster requirement in the primary *a priori* areas of evaluation (AOEs)** | | | | | |
| R. VTA/SN | 11.5 -19.2 -13.6 | | 17 | | 0.000407* |
| L. MFB/LNH | -9.5 -3.9 -11.8 | | 9 | | 0.004722† |
| **FA group differences which met the 27-voxel cluster requirement in the secondary *a priori* AOEs** | | | | | |
| R. ACC wm | | 19.2 32.7 -4.7 | | 35 | 0.000532† |
| R. DLPFC wm (superior fr. gyrus) | | 18.3 26.5 49.5 | | 27 | 0.000112* |
| L. DLPFC wm (superior fr. gyrus) | | -13.3 23.1 44.1 | | 55 | 0.000609† |
| L. DLPFC wm (middle fr. gyrus) | | -38.6 26.0 31.5 | | 40 | 0.000065* |
| L ACC/PAC wm | | -9.5 38.5 17.0 | | 33 | 0.000227† |
| **FA group differences which met the 81-voxel cluster requirement in other regions** | | | | | |
| L. PMC wm (SLF3) | | -43.9 0.0 18.9 | | 81 | 0.000027† |
| R. PMC wm (SLF3) | | 45.5 0.0 17.0 | | 134 | 0.000004* |
| L./midline CC | | -4.7 5.3 22.5 | | 98 | 0.001288 |
| R. CCtx wm | | 30.6 -57.5 2.6 | | 83 | 0.000001* |
| L. CCtx wm | | -21.0 -60.1 2.7 | | 99 | 0.000331 |
| * p value met the corrected threshold in the permutation test  † p value was within an order of magnitude of the corrected threshold (a trend) in the permutation test | | | | | |
| Abbreviations: R: right hemisphere; L: left hemisphere; wm: white matter; gm: gray matter; VTA/SN: ventral tegmental area/substantia nigra; MFB: medial forebrain bundle; LNH: lateral nucleus of the hypothalamus; ACC anterior cingulate cortex; DLPFC: dorsolateral prefrontal cortex; PAC: paracingulate cortex; PMC: premotor cortex; CC: corpus callosum; CCtx: calcarine cortex. | | | | | |

| **Table S2. Mean FA Values in Clusters from Voxel-Based Contrast for the Whole Cohort (22 MDD Versus 22 Matched Controls)** | | | |
| --- | --- | --- | --- |
| **Region** | **MDD mean (SD) FA across cluster** | | **Control mean (SD) FA across cluster** |
| **FA group differences which met the 9-voxel cluster requirement in the primary *a priori* areas of evaluation (AOEs)** | | | |
| R. VTA/SN* | 0.492 (0.134) | | 0.360 (0.0887) |
| L. MFB/LNH† | 0.265 (0.123) | | 0.392 (0.156) |
| **FA group differences which met the 27-voxel cluster requirement in the secondary *a priori* AOEs** | | | |
| R. ACC wm† | | 0.477(0.0671) | 0.562 (0.0855) |
| R. DLPFC wm (superior fr. gyrus) * | | 0.185 (0.0628) | 0.271 (0.0704) |
| L. DLPFC wm (superior fr. gyrus) † | | 0.261 (0.0935) | 0.363 (0.0882) |
| L. DLPFC wm (middle fr. gyrus) * | | 0.204 (0.0726) | 0.303 (0.0699) |
| L ACC/PAC wm† | | 0.289 (0.0720) | 0.379 (0.0760) |
| **FA group differences which met the 81-voxel cluster requirement in other regions** | | | |
| L. PMC wm (SLF3) † | | 0.268 (0.0748) | 0.389 (0.0877) |
| R. PMC wm (SLF3) * | | 0.316 (0.0767) | 0.414 (0.0447) |
| L./midline CC | | 0.557 (0.168) | 0.693 (0.0922) |
| R. CCtx wm* | | 0.517 (0.0557) | 0.395 (0.0958) |
| L. CCtx wm | | 0.432 (0.111) | 0.291 (0.124) |
| * p value met the corrected threshold in the permutation test  † p value was within an order of magnitude of the corrected threshold (a trend) in the permutation test | | | |
| Abbreviations: R: right hemisphere; L: left hemisphere; wm: white matter; gm: gray matter; VTA/SN: ventral tegmental area/substantia nigra; MFB: medial forebrain bundle; LNH: lateral nucleus of the hypothalamus; ACC anterior cingulate cortex; DLPFC: dorsolateral prefrontal cortex; PAC: paracingulate cortex; PMC: premotor cortex; CC: corpus callosum; CCtx: calcarine cortex. | | | |

| **Table S3. Permutation Test for ”Abnnormal VTA/SN” MDD Subgroup (10 MDD Versus 10 Matched Controls)** | | | | | |
| --- | --- | --- | --- | --- | --- |
| **Region** | **MNI coordinates at peak difference** | | **Cluster size**  **(# voxels)** | | **p value in permutation test** |
| **FA group differences which met the 9-voxel cluster requirement in the primary *a priori* areas of evaluation (AOEs)** | | | | | |
| R. VTA/SN | 11.4 -21.1 -11.9 | | 49 | | 0.000011* |
| L. VTA/SN | -5.7 -19.2 -17.2 | | 13 | | 0.003897† |
| **FA group differences which met the 27-voxel cluster requirement in the secondary *a priori* AOEs** | | | | | |
| L. DLPFC wm (superior fr. gyrus) | | -15 26.9 32.8 | | 50 | 0.003031 |
| L. DLPFC wm (middle fr. gyrus) | | -37.7 27.5 27.7 | | 33 | 0.000422† |
| **FA group differences which met the 81-voxel cluster requirement in other regions** | | | | | |
| R. FOC (olf sulcus) gm | | 9.5 17.9 -15.4 | | 95 | 0.000022† |
| R. SGC gm | | 7.6 10.7 -5.8 | | 118 | 0.001169 |
| R. PMC wm (SLF3) | | 53 9.2 15.2 | | 106 | 0.000043† |
| MDM/PAG region | | 0 -24.6 -7.2 | | 78 (just below threshold) | 0.001104 |
| * p value met the corrected threshold in the permutation test  † p value was within an order of magnitude of the corrected threshold (a trend) in the permutation test | | | | | |
| Abbreviations: R: right hemisphere; L: left hemisphere; wm: white matter; gm: gray matter; VTA/SN: ventral tegmental area/substantia nigra; DLPFC: dorsolateral prefrontal cortex; FOC: orbitofrontal cortex; SGC: subgenual cortex; PMC: premotor cortex; CCtx: calcarine cortex; MDM/PAG: medial dorsal midbrain/periaqueductal gray. | | | | | |

| **Table S4. Mean FA Values in Clusters from Voxel-Based Contrast for the “Abnormal VTA/SN” MDD Subgroup (10 MDD Versus 10 Matched Controls)** | | | |
| --- | --- | --- | --- |
| **Region** | **MDD mean (SD) FA across cluster** | | **Control mean (SD) FA across cluster** |
| **FA group differences which met the 9-voxel cluster requirement in the primary *a priori* areas of evaluation (AOEs)** | | | |
| R. VTA/SN* | 0.553 (0.0576) | | 0.342 (0.0759) |
| L. VTA/SN† | 0.516 (0.146) | | 0.316 (0.116) |
| **FA group differences which met the 27-voxel cluster requirement in the secondary *a priori* AOEs** | | | |
| L. DLPFC wm (superior fr. gyrus) | | 0.255 (0.108) | 0.402 (0.0725) |
| L. DLPFC wm (middle fr. gyrus) † | | 0.169 (0.0400) | 0.294 (0.0760) |
| **FA group differences which met the 81-voxel cluster requirement in other regions** | | | |
| R. FOC (olf sulcus) gm† | | 0.347 (0.0935) | 0.165 (0.0346) |
| R. SGC gm | | 0.370 (0.117) | 0.211 (0.0706) |
| R. PMC wm (SLF3) † | | 0.222 (0.0430) | 0.370 (0.0728) |
| MDM/PAG region | | 0.430 (0.0997) | 0.270 (0.0802) |
| * p value met the corrected threshold in the permutation test  † p value was within an order of magnitude of the corrected threshold (a trend) in the permutation test | | | |
| Abbreviations: R: right hemisphere; L: left hemisphere; wm: white matter; gm: gray matter; VTA/SN: ventral tegmental area/substantia nigra; DLPFC: dorsolateral prefrontal cortex; FOC: orbitofrontal cortex; SGC: subgenual cortex; PMC: premotor cortex; CCtx: calcarine cortex; MDM/PAG: medial dorsal midbrain/periaqueductal gray. | | | |

| **Table S5. Permutation Test for “Normal VTA/SN” MDD Subgroup (12 MDD Versus 12 Matched Controls)** | | | | | |
| --- | --- | --- | --- | --- | --- |
| **Region** | **MNI coordinates at peak difference** | | **Cluster size**  **(# voxels)** | | **p value in permutation test** |
| **FA group differences which met the 9-voxel cluster requirement in the primary *a priori* areas of evaluation (AOEs)** | | | | | |
| L. MFB/LNH | -11.3 -3.3 -9.8 | | 20 | | 0.005504† |
| **FA group differences which met the 27-voxel cluster requirement in the secondary *a priori* AOEs** | | | | | |
| R. ACC wm | | 15.2 31.6 -3.2 | | 31 | 0.000046* |
| L ACC/PAC wm | | -9.4 38 13.8 | | 46 | 0.001271 |
| * p value met the corrected threshold in the permutation test  † p value was within an order of magnitude of the corrected threshold (a trend) in the permutation test | | | | | |
| Abbreviations: R: right hemisphere; L: left hemisphere; wm: white matter; MFB: medial forebrain bundle; LNH: lateral nucleus of the hypothalamus; ACC anterior cingulate cortex; PAC: paracingulate cortex. | | | | | |

| **Table S6. Mean FA Values in Clusters from Voxel-Based Contrast for the “Normal VTA/SN” MDD Subgroup (12 MDD Versus 12 Matched Controls)** | | | |
| --- | --- | --- | --- |
| **Region** | **MDD mean (SD) FA across cluster** | | **Control mean (SD) FA across cluster** |
| **FA group differences which met the 9-voxel cluster requirement in the primary *a priori* areas of evaluation (AOEs)** | | | |
| L. MFB/LNH† | 0.275 (0.103) | | 0.440 (0.148) |
| **FA group differences which met the 27-voxel cluster requirement in the secondary *a priori* AOEs** | | | |
| R. ACC wm* | | 0.448 (0.0796) | 0.569 (0.0578) |
| L ACC/PAC wm | | 0.419 (0.0806) | 0.558 (0.0962) |
| * p value met the corrected threshold in the permutation test  † p value was within an order of magnitude of the corrected threshold (a trend) in the permutation test | | | |
| Abbreviations: R: right hemisphere; L: left hemisphere; wm: white matter; MFB: medial forebrain bundle; LNH: lateral nucleus of the hypothalamus; ACC anterior cingulate cortex; PAC: paracingulate cortex. | | | |
