# Supplementary material for: Microstructural Abnormalities in Subcortical Reward Circuitry of Subjects with Major Depressive Disorder
Source: PLoS One. 2010 Nov 29;5(11):e13945. doi: 10.1371/journal.pone.0013945 (PMC2993928; doi:10.1371/journal.pone.0013945)
Supplement: Dataset S2 — VT abnormalities and laterality. Analysis evaluating whether right hemisphere VT abnormalities in the MDD cohort reflected a loss of normal hemispheric asymmetry in this region or a bilateral change that was simply more significant in one hemisphere than the other. (0.03 MB DOC) [file pone.0013945.s010.doc]

**Supporting Information Dataset II (Dataset SII): VTA/SN Abnormalities and Laterality**

**A. Methods**

Because there were no group difference clusters detected in the left VTA/SN, we evaluated the likelihood that the right hemisphere VTA/SN abnormality represented an asymmetry of microstructure. To do this, we created a mirror image cluster in the same anatomical region in the left VTA/SN and used this to evaluate whether there was a significant left/right hemispheric difference in FA in the control and MDD cohorts. A permutation analysis was used to evaluate hemispheric differences, following the procedures described in Dataset SI.

We also tested laterality in the follow-up analyses. We created a mirror image cluster (using the cluster from the abnormal VTA/SN subgroup contrast) in the same anatomical region in the left VTA/SN and used this to evaluate whether there was a significant left/right hemispheric difference in FA in the abnormal VTA/SN MDD subgroup versus controls. We also evaluated left/right differences by comparing FA values from the left VTA/SN cluster detected in the subgroup analysis with FA values from the right hemisphere cluster. A permutation analysis was used to evaluate left/right differences, following the procedures described in Dataset SI.

**B. Results**

**1. Main analyses.** In contrast to the right VTA/SN findings, only three contiguous voxels at p<0.05 were observed within the left VTA/SN in the initial group contrast (i.e. this was below the cluster threshold). Despite this apparent laterality of the VTA/SN finding, a permutation test comparing values in the right VTA/SN cluster against values in a mirror image cluster in the left hemisphere indicated that FA in the VTA/SN region did not differ significantly across the two hemispheres in the MDD cohort (p=0.45). The control cohort showed a trend toward a hemispheric difference in this region, with lower FA in the left than the right VTA/SN (p=0.065).

**2. Follow-up analyses.** There were no hemispheric differences in VTA/SN FA in either the MDD or the control cohorts included in the abnormal VTA/SN subgroup contrast (MDD: p=0.66 for mirror image of right hemisphere cluster, p=0.34 for statistically defined cluster; control: p=0.26 for mirror image of right hemisphere cluster, p=0.34 for statistically defined cluster).

**C. Discussion/Interpretation**

There were no hemispheric differences in the VTA/SN region in the abnormal VTA/SN subgroup, suggesting our findings did not indicate a hemispheric asymmetry in MDD subjects. However, there was a trend toward hemispheric differences in control subjects in the whole-cohort VTA/SN comparison, so it cannot be ruled out that at least part of the change in patients might have reflected a loss of normal asymmetry in SN microstructure.
